# In vivo expression of VCAM1 precedes nephron loss following kidney tubular necrosis

**DOI:** 10.1126/sciadv.adz5358

**Published:** 2025-10-22

**Authors:** Anders M. Kristensen, Luca Bordoni, Marie B. Nielsen, Anna Faivre, Hanne Kidmose, Donato Sardella, Katherine E. Shipman, Nicoline V. Krogstrup, Joachim Størling, Henrik Birn, Rune Enger, Ina Maria Schiessl

**Affiliations:** ^1^ Department of Biomedicine, Aarhus University, Aarhus, Denmark.; ^2^ Letten Centre, Department of Molecular Medicine, Institute of Basic Medical Sciences, University of Oslo, Oslo, Norway.; ^3^ Department of Clinical Medicine, Aarhus University, Aarhus, Denmark.; ^4^ Department of Renal Medicine, Aarhus University Hospital, Aarhus, Denmark.; ^5^ School of Medicine, University of Pittsburgh, Pittsburgh, USA.; ^6^ Steno Diabetes Center Copenhagen, Copenhagen, Denmark.; ^7^ Department of Neurosurgery, Oslo University Hospital, Rikshospitalet, Oslo, Norway.; ^8^ K. G. Jebsen Centre for Brain Fluid Research, Oslo, Norway.; ^9^ Department of Medicine 2, RWTH Aachen University, Aachen, Germany.

## Abstract

Nephron loss is a key event during onset and progression of chronic kidney disease, yet the mechanisms dictating tubule repair versus atrophy remain poorly understood. While fibrosis has been proposed to drive progressive organ damage, antifibrotic therapies have failed in clinical trials. Here, we reveal that tubular vascular cell adhesion molecule 1 (VCAM1) expression precedes nephron loss, fibrosis, and long-term kidney dysfunction. Using serial intravital microscopy in transgenic mice, we track tubulointerstitial remodeling between injured and intact tissue over 3 weeks. VCAM1 is rapidly induced in a distinct subset of injured tubules, preceding atrophy with sustained fibroblast recruitment. However, fibroblasts remain confined to injury sites and do not cause secondary damage in uninjured tubules. Last, in human kidney transplant biopsies, tubular VCAM1 expression, but not kidney injury molecule 1, correlates negatively with early and 12-month graft function, underscoring its potential as a biomarker of adverse outcomes. These findings position VCAM1 as an early indicator of tubular fate and nephron loss.

## Teaser

Serial imaging of the same kidney cells demonstrates tubular VCAM1 as an early indicator of nephron loss and kidney dysfunction.

## INTRODUCTION

Nephron loss is a key event in the onset and progression of chronic kidney disease (CKD), and preventing nephron loss is a major strategy for preserving kidney function. Acute tubular necrosis is a common cause of acute kidney injury (AKI) (*1*) and may transition into CKD upon failed tubule repair and fibrosis (*2*–*5*). Females are less sensitive to AKI, and estradiol functions inhibit ferroptosis (*6*). However, the decision between successful tubule repair or atrophy likely occurs downstream of tubule necrosis, and the mechanisms behind this decision process remain poorly understood (*7*). Tubular necrosis is followed by luminal accumulation of necrotic debris, which has been associated with propagation of injury, extending damage beyond the initial sites of necrosis (*8*). At the same time, a late-emerging population of injured tubule cells has been characterized demonstrating de novo expression of vascular cell adhesion molecule 1 (VCAM1) (*9*–*11*), which selectively marks atrophic and nonrecovering tubules at late stages after AKI (*8*). Molecularly, this population displays a proinflammatory and profibrotic gene signature indicative of failed tubule repair (*9*–*11*) and is spatially associated with fibrotic niches (*12*, *13*). Furthermore, fibrosis has been suggested to determine nephron fate. Persistent fibrosis is a hallmark of AKI-CKD transition (*14*), and the extent of tubulointerstitial fibrosis in CKD correlates negatively with residual kidney function (*15*). Considered a pathological extension of wound healing (*15*, *16*), fibrosis may determine the decision process between successful and failed repair after tubule necrosis through continuous tissue scarring (*5*) and tubular constriction (*17*). Furthermore, fibrosis may affect the fate of uninjured nephrons in close proximity to fibrotic sites and drive kidney disease progression through self-sustained deposition of extracellular matrix (ECM) (*18*), causing secondary tubular injury (*15*) and new fibrotic foci (*19*–*21*). Nevertheless, antifibrotic drugs have shown limited efficacy to halt CKD in clinical trials (*20*, *22*), and several studies have pointed toward potential proregenerative actions of (myo)fibroblasts (*23*–*26*).

Emerging cells of failed tubule repair and fibrosis development may ultimately orchestrate the decision between nephron loss or survival. However, because of limited longitudinal data, the dynamics of tubulointerstitial remodeling in relation to nephron fate remain incompletely understood, and several key questions are yet to be answered: Which tubule segments undergo failed repair? Can VCAM1-positive tubules recover, or do they inevitably progress toward atrophy? Does fibrosis determine nephron fate upon injury? Can fibrosis drive progressive kidney disease by inducing secondary injury in the surrounding tissue?

In this study, we aimed to shed light on the above questions and elucidate the spatiotemporal dynamics underlying nephron loss. Using serial intravital microscopy of transgenic mice, we tracked the interactions of renal fibroblasts and pericytes with injured and uninjured tubules over time and tested whether fibrosis preceded secondary tubular damage in uninjured tissue. To achieve this, we used a model of partial ischemia-reperfusion injury (partial IRI) (*8*), which resulted in rapid and abundant tubule atrophy and fibrosis in approximately half of the kidney while demonstrating uninjured renal tissue in the other half. To determine the impact of fibrosis on the survival of injured and uninjured tubule segments, we applied systematic spatiotemporal mapping of fibroblast recruitment relative to tubule necrosis, remodeling, and atrophy development. Using correlative imaging, we deciphered the structural and functional attributes of early VCAM1-expressing tubules and linked these to nephron fate.

Our data emphasize a decisive role of tubular injury for nephron loss. We demonstrate that VCAM1 positivity is rapidly induced in a distinct subset of severely injured tubules and precedes nephron loss. Concomitantly, VCAM1-positive tubules demonstrated enhanced fibroblast recruitment, and the extent of fibroblast recruitment toward an injured tubule was positively associated with the ultimate development of tubule atrophy. In contrast, fibroblast recruitment toward uninjured tubules was not associated with a higher risk of atrophy and seemed protective against later manifestations of tubule injury. Last, and consistent with a key role of tubule atrophy for nephron loss and AKI-CKD transition, we demonstrate a negative correlation of tubular VCAM1, but not kidney injury molecule 1 (KIM1), in day 6 biopsies with early and 1-year graft function upon human kidney transplantation.

## RESULTS

### Partial IRI results in abundant tubule atrophy in postischemic tissue

**Fig. 1.**
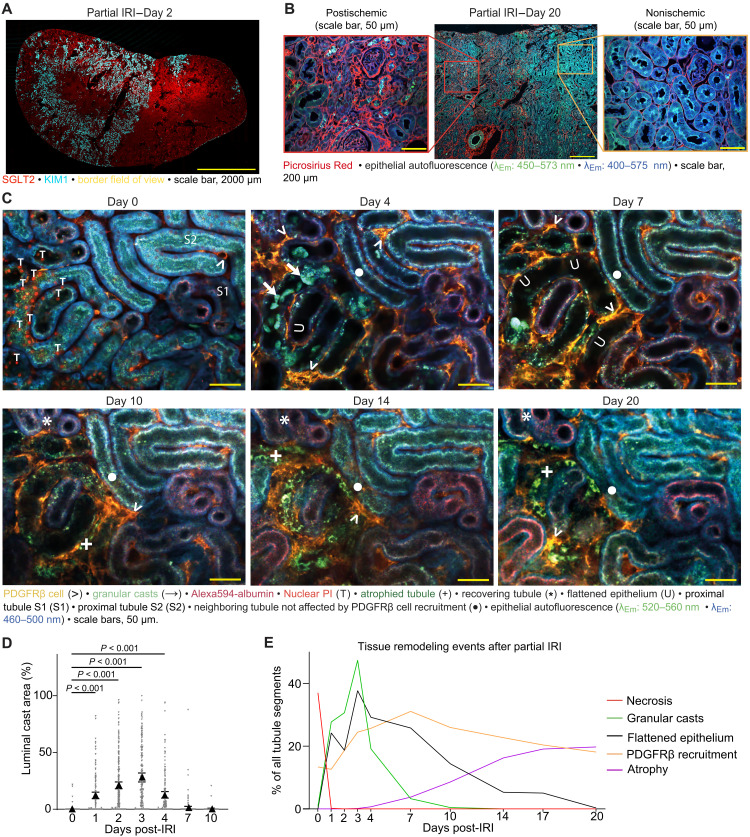
Tubule remodeling upon partial IRI. (**A**) Representative KIM1 (cyan) and SGLT2 (red) immunostaining of a day 2 partial IRI kidney demonstrating tubule injury in approximately half of the kidney. In vivo serial imaging was conducted at the border between the ischemic and nonischemic areas. (**B**) Representative Picrosirius Red staining of a day 20 partial IRI kidney demonstrates atrophy and fibrosis in the postischemic tissue. (**C**) Serial in vivo two-photon microscopy of a border region between postischemic and nonischemic area of a PDGFRβ-tdTomato reporter mouse kidney on days 0, 4, 7, 10, 14, and 20 post–partial IRI demonstrates tubular necrosis on day 0 [propidium iodide (PI), T], which is followed by luminal granular cast accumulation (→), flattening of the tubule epithelium (U), and, lastly, tubule atrophy (+). In contrast, some tubules displaying less injury undergo recovery (*). Note the PDGFRβ cell (>) accumulation around injured tubules, which peaks at day 7 and gradually resolves thereafter. (**D**) Quantification of luminal granular cast accumulation in damaged tubules over time (*n* = 286 tubule segments from five male and five female partial IRI mice). Statistical test: linear mixed-effects model, *P* values from two-sided tests (data S1, a). (**E**) Timeline of tissue remodeling events after partial IRI.

To investigate interstitial cell remodeling in injured and noninjured tissue, we performed partial IRI (*8*) in transgenic platelet-derived growth factor receptor β (PDGFRβ)–Cre recombinase-Estrogen Receptor T2 (CreERT2) (*27*)–Salsa6F (*28*) (PDGFRβ-tdTomato) mice, which identify renal fibroblasts and pericytes by tdTomato expression. Occluding only one branch of the renal artery for 21 min during the partial IRI procedure rendered approximately half of the kidney ischemic, while the other half remained perfused. Partial IRI mice demonstrated albuminuria on day 1 (fig. S1A), reduced sodium glucose transporter 2 (SGLT2) expression with pronounced KIM1 up-regulation on day 2 (Fig. 1A), and extensive tubule atrophy and fibrosis in postischemic tissue regions on day 20 (Fig. 1B). Glomerular filtration rate (GFR) remained stable over time (fig. S1B).

Careful implantation and placement of winged abdominal imaging windows (wAIWs) (*29*) above partial IRI kidneys (*8*) allowed tracking of PDGFRβ cells in the border region of postischemic and nonischemic renal tissue over time using serial in vivo two-photon microscopy. Upon reperfusion, postischemic tissue could be identified on the basis of nuclear propidium iodide (PI) staining in necrotic tubule cells (Fig. 1C, day 0). At 2 hours after reperfusion, 37% of the tubule segments located in the border regions of the partial IRI kidneys displayed necrosis with an average of 39 ± 2% necrotic cells per total segment nuclei (*n* = 752 tubule segments from 10 mice). Notably, PI staining was almost exclusively detectable at day 0 and revealed a weak correlation with atrophy development (*r*^2^ = 0.07; fig. S1, C to F). Furthermore, we observed substantial accumulation of granular casts in the lumen of proximal tubules, which was most prominent in the first 4 days after partial IRI. At day 7, only 3% of all partial IRI tubule segments demonstrated granular casts, and at day 10, granular casts were exclusively observed in distal convoluted tubules and collecting ducts (Fig. 1, C to E). Granular casts were detectable not only in the lumen of necrotic tubules but also in initially nonnecrotic tubules, supporting upstream necrotic injury with subsequent downstream accumulation of cellular debris (Fig. 3A), as previously demonstrated (*8*). From day 1, tubule injury resulted in a persistent and characteristic flattening of the tubule epithelium, which was evident in 24% of all partial IRI tubule segments at day 1, in 26% at day 7, and then gradually decreased to 5% at day 17 (*n* = 752 tubule segments from 10 mice; Fig. 1E).

**Fig. 2.**
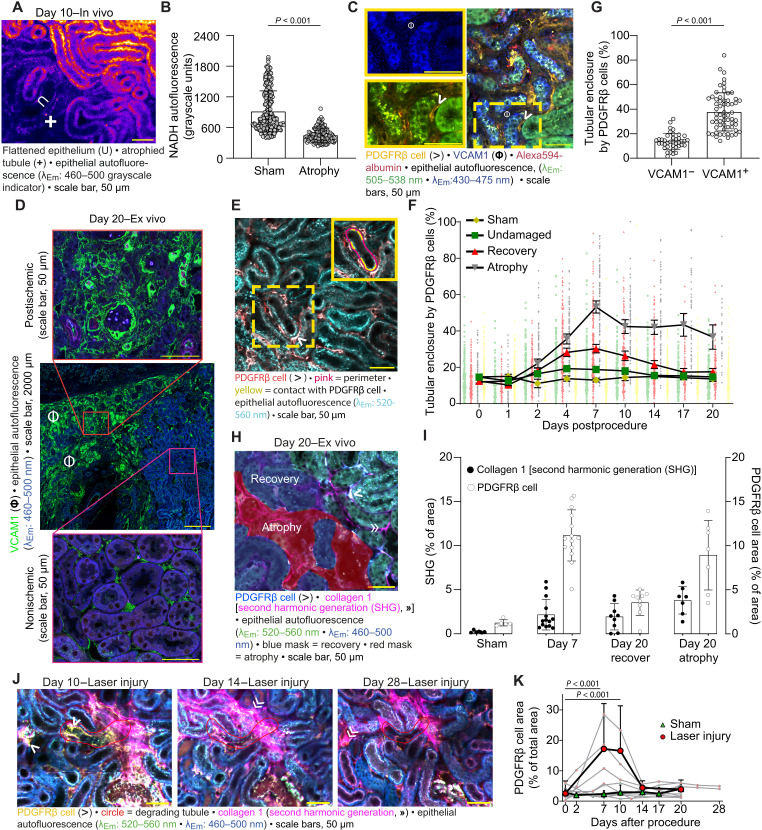
Sustained PDGFRβ cell recruitment and fibrosis around atrophic tubules. (**A**) NADH autofluorescence (λ_Ex_ = 750 nm) indicated in warm/cold colors for high/low fluorescence intensity. (**B**) Quantification of epithelial NADH autofluorescence in sham (*n* = 266; two males and one female) and atrophic tubules (*n* = 126; three males and four females). Means ± 95% confidence interval (CI) with scatterplot. Linear mixed-effects model, two-sided *P* values versus sham (data S1, b). (**C**) Ex vivo immunostaining reveals VCAM1 positivity (Φ) in atrophic tubules with PDGFRβ cell enclosure (>). (**D**) Overview of VCAM1 immunostaining on partial IRI kidney. (**E**) Enclosure was calculated as the percentage of tubule perimeter in contact with PDGFRβ cells (inset: yellow versus pink). (**F**) Quantification of PDGFRβ cell enclosure of sham (*n* = 250; two males and one female) and partial IRI (five males and five females) tubules, categorized as undamaged (*n* = 225), recovery (*n* = 233), and atrophy (*n* = 142). Statistics: fig. S5 (data S1, c). (**G**) Quantification of PDGFRβ cell enclosure of VCAM1-negative (*n* = 40) and VCAM1-positive (*n* = 62) tubules (two males and one female). Means ± 95% CI with scatterplot. Linear mixed-effects model, two-sided *P* values (data S1, d). (**H**) Representative masks of recovered (blue) and atrophic (red) tubules. (**I**) Quantification of segmented second harmonic generation (SHG) signal and PDGFRβ cells from sham (*n* = 6; two males and one female), day 7 (*n* = 14; two males and two females), and day 20 partial IRI kidneys (one male and two females) showing recovered (*n* = 9) and atrophic areas (*n* = 7). Means ± 95% CI with scatterplot. Statistics: fig. S6 (G and H) (data S1, e). (**J**) Serial in vivo two-photon microscopy of a PDGFRβ-tdTomato kidney after focal laser injury. Red circle indicates injured tubule undergoing atrophy (day 14) and degradation (day 28). (**K**) Quantification of segmented PDGFRβ cells in sham (*n* = 3; two males and one female) and laser injury fields of view (FOVs) (*n* = 4; one male and three females). Means ± 95% CI with scatterplot. Linear mixed-effects model, two-sided *P* values versus baseline (data S1, f).

By day 20, injured and remodeling tubules had either undergone successful recovery or transitioned into a collapsed nonreversible state, identified as tubule atrophy (Fig. 1E) (*8*). Throughout the observation period, 24% of all tubule segments in the border regions atrophied, 20% recovered, and 37% did not show any structural or functional indication of damage and were categorized as undamaged. In the remaining 19%, determination of the final fate was not possible because of detaching of the wAIW before day 20 (tables S1 and S2). On day 0, both recovering and atrophic S1 proximal tubules (PT-S1) in partial IRI kidneys revealed significantly reduced albumin reuptake capacity as compared to uninjured partial IRI and sham PT-S1 segments. While albumin reuptake capacity in recovering tubules gradually recovered by day 7, it remained reduced throughout the end of the observation period in atrophic tubules (fig. S1G). Furthermore, atrophic tubules also exhibited strongly decreased 1,4-dihydronicotinamide adenine dinucleotide (NADH) autofluorescence [excitation wavelength (λ_Ex_) = 750 nm and emission wavelength (λ_Em_) = 460 - 500 nm] (*30*–*32*) as compared to sham tubule segments (*P* < 0.001; Fig. 2, A and B). Last, ex vivo immunostaining of fixed partial IRI kidney tissue at day 20 confirmed selective staining for the profibrotic marker VCAM1 (*10*, *33*) in atrophic tubules (Fig. 2C), with vast VCAM1 expression and atrophy development in the postischemic tissue (Fig. 2D).

### PDGFRβ cell recruitment only persists around atrophic tubule segments

Tubular remodeling in partial IRI kidneys was associated with increasing macrophage infiltration over time (fig. S2A) and pronounced PDGFRβ cell expansion (fig. S2B). To assess dynamic PDGFRβ cell expansion, we first used machine learning–based image segmentation (*34*, *35*) of tdTomato-positive PDGFRβ cells in the in vivo two-photon microscopy images (fig. S2C) and quantified their abundance at each imaging time point (fig. S2D). We observed a progressive increase in the relative PDGFRβ cell area from 2.1 ± 0.3% at day 0 to 7.3 ± 1.3% at day 7, which was followed by a gradual decrease while remaining elevated compared to baseline (*P* = 0.039 for day 20 versus day 0; fig. S2D). In contrast, no significant changes in PDGFRβ cell area were observed in sham mice. To determine whether the observed expansion of PDGFRβ-positive cells resulted from proliferation, we performed partial IRI in transgenic cyclin B1 (CycB1)–green fluorescent protein (GFP) reporter (*36*) mice, which mark cells in the S-G_2_-M phases of the cell cycle through transient GFP expression. We then stained for PDGFRβ and assessed the number of GFP-positive PDGFRβ and tubule cells, respectively. Control CycB1 kidneys revealed no detectable GFP positivity in PDGFRβ cells, while tubules occasionally expressed GFP (fig. S3A). On day 3 after partial IRI, the number of GFP-positive PDGFRβ and tubule cells increased significantly (*P* < 0.001), with proliferating PDGFRβ cells mostly located around damaged and proliferating tubules. By day 14, proliferation of PDGFRβ and tubule cells had ceased and was not different from control (fig. S3, B and C). These results demonstrate the transient proliferation of PDGFRβ cells upon partial IRI that largely concentrated around proliferating tubules.

To further confirm whether PDGFRβ cells accumulated mostly around injured tubules, we next assessed the enclosure of a given tubule segment by PDGFRβ cells by measuring the relative extent of the epithelial cell–cell contact with adjacent PDGFRβ cells expressed as a percentage of the total tubule epithelial perimeter (Fig. 2E). Tubule segments were defined as injured if they presented at least one of the following criteria on any of the imaging days: PI positivity, luminal granular cast accumulation, flattened tubule epithelium, dilated or collapsed tubular lumen, visually decreased NADH autofluorescence, visually reduced albumin reuptake in PT-S1 segments, and atrophy. Enclosure of injured tubules by PDGFRβ cells was twofold higher than that of uninjured tubules (*P* < 0.001; fig. S4). Next, we categorized individual tubule segments as sham, undamaged, recovered, or atrophic and plotted the enclosure by PDGFRβ cells over time (Fig. 2F and fig. S5, A and B). Enclosure of sham tubule segments by PDGFRβ cells remained stable across all time points, while the enclosure of recovering tubules increased twofold by day 7 (*P* < 0.001) and then reversed by day 14. In contrast, the enclosure of atrophic tubules by PDGFRβ cells increased fourfold by day 7 (*P* < 0.001) and then gradually decreased to twofold levels of baseline by day 20 (*P* < 0.001). Consistent with profibrotic properties of VCAM1 (*10*, *37*), ex vivo analysis of VCAM1-stained kidneys at day 20 confirmed significantly higher PDGFRβ cell enclosure of VCAM1-positive (atrophic) tubules compared to VCAM1-negative (nonatrophic) tubules (*P* < 0.001; Fig. 2, C and G). PDGFRβ cell enclosure around undamaged tubule segments was modestly elevated on days 4 and 7 and reversed to sham levels by day 10 (Fig. 2F and fig. S5, A and B). In summary, these results demonstrate that recruitment of PDGFRβ cells upon partial IRI is reversible around recovering tubules but sustains around atrophic tubules.

We further investigated whether PDGFRβ cell accumulation in partial IRI kidneys was also associated with interstitial fibrosis. Coimmunostaining against α–smooth muscle actin (αSMA) and PDGFRβ on partial IRI kidneys confirmed abundant differentiation of PDGFRβ cells into myofibroblasts at days 4 and 7. In contrast, PDGFRβ cells at day 20 were largely αSMA negative, indicating redifferentiation (fig. S6, A and C). Alongside myofibroblast differentiation, Picrosirius Red staining indicated a fivefold increase in fibrosis in the postischemic tissue of day 20 partial IRI kidneys over respective levels in nonischemic tissue (fig. S6, B and D). Collagen I up-regulation and deposition are hallmarks of fibrosis (*38*) and can be detected in vivo via two-photon imaging of second harmonic generation (SHG) signal (*39*, *40*). To investigate the spatiotemporal dynamics of collagen deposition and PDGFRβ cell recruitment in more detail, we quantified the relative density of SHG signal and PDGFRβ cells in regions of remodeling tubules at day 7 and recovered and atrophic tubules of day 20 partial IRI kidneys. On the basis of serial imaging data, we identified recovered and atrophic tubules as previously injured tubules that had either readopted structurally intact morphology or atrophied by day 20 (Fig. 2H). Compared to the sham group, the SHG signal was significantly up-regulated around remodeling tubules at day 7 and around both recovered and atrophic tubules at day 20. However, while the SHG signal further increased around atrophic tubules from day 7 to day 20, it remained stable around recovered tubules (Fig. 2I and fig. S6G). This suggests that collagen deposition ceases following successful tubule repair but continues to increase around atrophic tubules. Consistently, we observed a marked increase in the abundance of PDGFRβ-positive cells around remodeling tubules at day 7 (*P* < 0.001 compared to sham), which, by day 20, had returned to sham levels around recovered tubules (*P* = 0.11) but remained elevated around atrophic tubules (Fig. 2I and fig. S6H). These data demonstrate that PDGFRβ cell recruitment is accompanied by interstitial fibrosis, with collagen deposition increasing in proportion to the duration of the PDGFRβ cell presence in the remodeling tissue.

### Atrophic tubules eventually degrade and disappear

Since kidney failure biopsies often show complete replacement of tubule epithelium with scar tissue (*14*) and the presence of atubular glomeruli (*41*–*43*), we hypothesized that atrophic tubules would eventually degrade and disappear. Upon partial IRI, our observation period did not suffice to document tubule degradation because tissue shrinkage associated with atrophy development impaired image quality or resulted in detachment of the kidney from the wAIW. Therefore, we applied a spatially restricted injury model and facilitated focal laser injury through focused two-photon laser exposure in healthy PDGFRβ-tdTomato kidneys (*n* = 4). Tissue remodeling in the wound was followed for up to 4 weeks using serial in vivo imaging (fig. S7A) and demonstrated similar patterns as observed after partial IRI. In the first week, tubules surrounding the laser ablation revealed luminal granular cast accumulation and flattened epithelial morphology, which was followed by atrophy development and VCAM1 positivity in the second week after focal laser injury (fig. S7B). Longitudinal imaging after laser injury further revealed the complete degradation of ~25% of the atrophic tubule segments (10 of 38 segments from four mice) (Fig. 2J and fig. S8), as well as the deposition of interstitial collagen around the remnant epithelium (Fig. 2J). Concomitantly with tubule atrophy and degradation, we also captured the closure of the wound as uninjured tubules adjacent to the atrophic nephrons gradually moved closer together (fig. S9). PDGFRβ cell recruitment after laser injury revealed a transient increase, peaking at day 7 and gradually reversing to baseline levels thereafter (Fig. 2K). Notably, PDGFRβ cells did not expand outside of the wound. In summary, atrophic tubules eventually undergo degradation, which is associated with wound closure and full reversibility of PDGFRβ cell recruitment.

### PDGFRβ cells do not expand into uninjured tissue regions over time

**Fig. 3.**
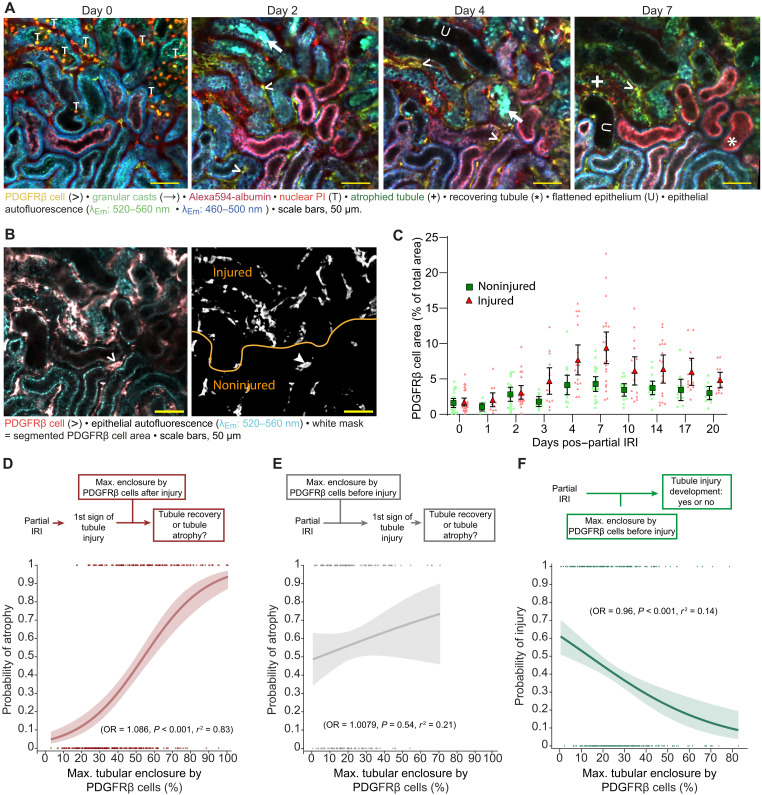
The impact of PDGFRβ cells on tubule fate. (**A**) Serial in vivo two-photon microscopy of a PDGFRβ-tdTomato reporter mouse kidney on days 0, 2, 4, and 7 post–partial IRI demonstrating a representative border region between nonischemic and postischemic tissue with spatially distinct areas of noninjured and injured tubules. Image segmentation (**B**) and quantification of segmented PDGFRβ cells (**C**) in masked areas of noninjured (*n* = 22) and injured tubule segments (*n* = 29) as categorized on the basis of serial imaging data from five male and five female partial IRI mice. Data are shown as means ± 95% CI. Statistical differences between different days and groups are indicated in fig. S10 (data S1, g). (**D**) Binary regression analysis of tubule fate (0 = nonatrophy and 1 = atrophy) and the respective maximum tubule enclosure by PDGFRβ cells after the first detection of tubular injury (*n* = 512 tubules from five male and five female mice). (**E**) Binary regression analysis of tubule fate (0 = nonatrophy and 1 = atrophy) and the respective maximum tubule enclosure by PDGFRβ cells before the first detection of tubule injury (*n* = 512 tubules from five male and five female mice). (**F**) Binary regression analysis of tubule fate (0 = noninjury and 1 = injury) and the respective maximum tubule enclosure by PDGFRβ cells before the first detection of tubule injury, if detectable (*n* = 470 tubules from five male and five female mice). *r*^2^ and effect size as odds ratio (OR) for the predictor of the binary regressions shown in (D) to (F) are reported in data S1 (h, i, and j), respectively. Tubular segments were classified as “injured” if they exhibited any of the following: positive staining for PI, dilated/collapsed tubule lumen, luminal granular cast accumulation, flattened tubule epithelium, visually decreased NADH autofluorescence, visually reduced albumin endocytosis in PT-S1, and/or atrophy.

To address whether fibrosis may spread toward uninjured tissue regions over time, we first evaluated the dynamic spatiotemporal distribution of PDGFRβ cells across injured and uninjured tissue. Each imaging field of view (FOV) was dissected into two different regions: an injured region (tubules displaying PI staining/luminal granular cast accumulation/flattened epithelium/ dilated or collapsed tubule lumen/decreased tubular NADH autofluorescence/reduced albumin endocytosis in PT-S1/atrophy) and an undamaged region with morphologically intact tubules, respectively (Fig. 3, A and B). To examine whether PDGFRβ cells eventually accumulated within previously noninjured tissue areas, we segmented PDGFRβ cells in each region and assessed their cell density over time (Fig. 3, B and C, and fig. S10). In the injured tissue region, PDGFRβ cell density was strongly increased from day 3, peaked on day 7, and then gradually decreased while remaining elevated over baseline. In contrast, we detected no PDGFRβ cell accumulation in uninjured tissue regions: Morphologically intact regions revealed a subtle but significant elevation of PDGFRβ cell density on day 4 (*P* = 0.037) and day 7 (*P* = 0.025), which returned to baseline levels by day 10 and remained low thereafter (Fig. 3C and fig. S10).

### Impact of PDGFRβ cell recruitment on tubule fate

Since PDGFRβ cells accumulated predominantly around atrophic tubules, we first tested whether increased exposure of an injured tubule to PDGFRβ cells increased its likelihood of atrophy. We plotted the maximum enclosure of a given tubule by PDGFRβ cells (as a percentage of the total tubule perimeter) determined after the first detection of tubule injury (independent variable) against the categorical outcome of the tubule (atrophy = 1/nonatrophy = 0, target variable), (Fig. 3E). This analysis revealed a significant relationship [*P* < 0.001, *r*^2^ = 0.833, odds ratio (OR) = 1.086; Fig. 3D], indicating that the risk of atrophy in injured tubules increased with higher PDGFRβ cell recruitment.

We next investigated the impact of PDGFRβ cells on uninjured tissue: Although PDGFRβ cells did not infiltrate into uninjured tissue regions over time, injury-adjacent tubules were still transiently exposed to higher levels of PDGFRβ cells (Fig. 3C and fig. S10). Therefore, we systematically determined whether increased exposure of an initially uninjured tubule to PDGFRβ cells increased its likelihood of atrophy. We plotted the maximum enclosure of a given tubule by PDGFRβ cells (as a percentage of the total tubule perimeter) determined before the first detection of tubule injury against the categorical outcome of the tubule (atrophy = 1/nonatrophy = 0) (Fig. 3E). Binary logistic regression analysis revealed no significant relationship, suggesting that increased PDGFRβ cell accumulation around an uninjured tubule does not increase its likelihood of atrophy.

Last, we determined whether increased exposure of an initially uninjured tubule to PDGFRβ cells was linked to secondary tubule injury at later time points. Thus, we determined the maximum enclosure of each partial IRI tubule segment by PDGFRβ cells during uninjured conditions and plotted these against the subsequent categorical outcome of the tubule as either injury (=1) or lack of injury (=0). Binary logistic regression analysis revealed a negative relationship between the extent of PDGFRβ cell enclosure around an uninjured tubule and the odds of later manifestations of injury (*P* < 0.001, *r*^2^ = 0.14, OR = 0.96; Fig. 3F). This suggested that PDGFRβ cells do not cause secondary tubule injury.

In summary, these analyses indicate a decisive role of tubule injury for nephron fate. While PDGFRβ cell recruitment predicted tubule atrophy among injured tubules, there was no significant relationship between PDGFRβ cell recruitment and the likelihood of atrophy among uninjured tubules. In contrast, PDGFRβ cell accumulation around uninjured tubules seemed protective against later manifestations of tubule injury.

### Early tubular VCAM1 expression precedes tubule atrophy

**Fig. 4.**
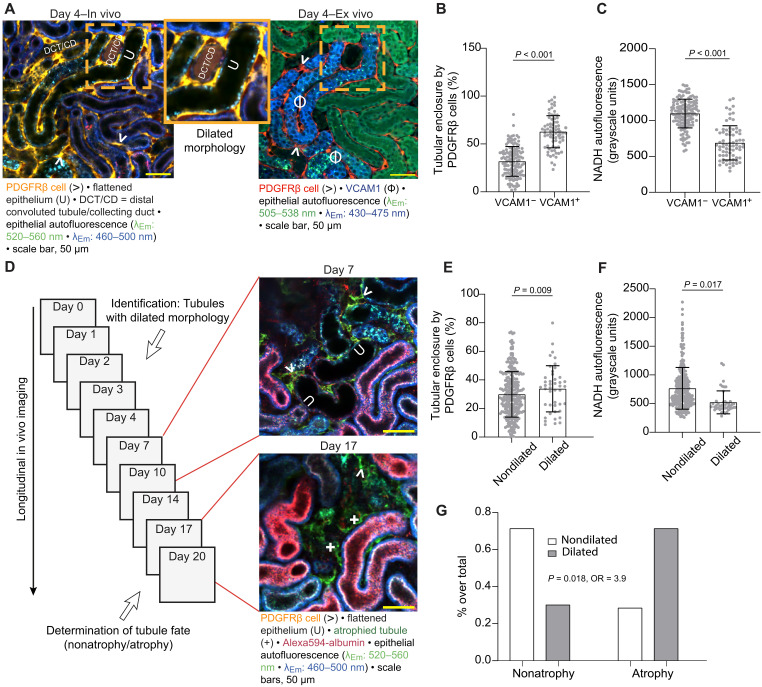
Early tubular VCAM1 expression increases the odds of tubule atrophy. (**A**) Correlative in vivo and ex vivo two-photon microscopy of the same kidney tissue acquired 4 days after partial IRI reveals VCAM1 expression (Φ) in a distinct population of injured tubules with flattened epithelium and dilated tubular lumen (dilated morphology, U). DCT/CD, distal convoluted tubule or collecting duct. (**B**) In vivo tubule enclosure by PDGFRβ cells and (**C**) epithelial NADH autofluorescence in VCAM1-negative and VCAM1-positive tubule segments at day 4 after partial IRI (identified via correlative microscopy as exemplified in A) (*n* = 209 tubule segments from three female mice). Means ± 95% CI with scatterplot. Statistical test: linear mixed-effects model, *P* values from two-sided tests (data S1, k and l). (**D**) Exemplified identification of tubules with dilated morphology and determination of the respective tubule fate from longitudinal imaging data: Representative serial in vivo two-photon microscopy of a PDGFRβ-tdTomato partial IRI kidney reveals tubules of dilated morphology at day 7 (U) that underwent atrophy by day 17 (+). (**E**) In vivo tubule enclosure by PDGFRβ cells and (**F**) epithelial NADH autofluorescence of tubules with and without dilated morphology identified on day 4 after partial IRI (*n* = 338 tubule segments from three male and four female mice). Means ± 95% CI with scatterplot. Statistical test: linear mixed-effects model, *P* values from two-sided tests (data S1, m and n). (**G**) Logistic regression of injured tubule segments with or without dilated morphology. Outcome: tubule atrophy as binary classifier. Predictor: tubule morphology of injured tubule with categorical classification of nondilated or dilated (*n* = 512 tubule segments from five male and five female mice). *r*^2^ and effect size as OR for the predictors are reported (data S1, o).

We recently showed that atrophic tubules selectively express VCAM1 (*8*) (Fig. 2C), a marker of failed tubule repair with profibrotic properties (*10*, *37*). While our data indicated the first appearance of atrophic tubules by day 7 after partial IRI (Fig. 1E), published single-cell RNA sequencing (scRNA-seq) data revealed tubular VCAM1 expression as early as 2 days after IRI (*10*). To access structural and functional attributes of early VCAM1-expressing tubules, we first tracked injured tubules in vivo using serial imaging and then tested for VCAM1 positivity in day 4 partial IRI kidneys using ex vivo correlative microscopy. These data revealed VCAM1 expression in a nonatrophic tubule population that identified with flattened tubule epithelium alongside dilated tubular lumen (defined as “dilated morphology”; Fig. 4A). Among all VCAM1-positive tubules (*n* = 74) at day 4, 52.7% demonstrated dilated morphology, 25.7% a precollapsed state with an almost or fully collapsed tubule lumen, and 17.6% dedifferentiated epithelium without pronounced dilation (fig. S11, A and B). Consistent with VCAM1’s profibrotic properties (*10*), day 4 VCAM1-positive tubules demonstrated higher enclosure by PDGFRβ cells than VCAM1-negative tubules (Fig. 4B). Furthermore, VCAM1-positive tubules demonstrated decreased NADH autofluorescence (Fig. 4C), indicative of metabolic dysfunction and NADH autofluorescence negatively correlated with the intensity of VCAM1 signal in the respective tubule (fig. S11C). CKD has been associated with metabolic dysregulation such as decreased rates of gluconeogenesis (*44*) and β-oxidation (*45*), which could contribute to decreased cellular levels of NADH (*46*). To validate that decreased NADH fluorescence in VCAM1-postive tubules derives from metabolic dysregulation, we analyzed a publicly available IRI single-nucleus RNA-seq dataset (*44*, *47*) to investigate healthy, injured, and failed repair (Vcam1-positive) proximal tubule cell clusters (fig. S11, D to F). This demonstrated the down-regulation of genes involved in gluconeogenesis and β-oxidation, such as Phosphoenolpyruvate carboxykinase (Pck1), fructose-1,6-bisphosphatase 1 (Fbp1) and Carnitine Palmitoyltransferase (Cpt1a), and fatty acid–binding protein 5 in Vcam1-positive cells (fig. S11G). In contrast, several profibrotic genes were up-regulated, including intercellular adhesion molecule–1 (Icam1) (fig. S11G), which has been recently proposed as a key determinant of the profibrotic phenotype in failed repair cells (*48*). Last, immunostainings on partial IRI kidneys revealed a decreased expression of PCK1 in VCAM1-positive tubules as compared to VCAM1-negative tubules (fig. S11H), indicating that a similar metabolic shift is also observable in the partial IRI model.

Since atrophic tubules at day 20 also demonstrated VCAM1 positivity (Fig. 2C) with decreased NADH autofluorescence (Fig. 2, A and B) and increased enclosure by PDGFRβ cells (Fig. 2G), we hypothesized that early VCAM1-expressing tubules would eventually turn atrophic. To test this hypothesis, we identified the early VCAM1-positive tubule population in the longitudinal partial IRI dataset based on its dilated morphology (Fig. 4D). Injured tubules of dilated morphology also demonstrated higher enclosure by PDGFRβ cells and lower epithelial NADH autofluorescence when compared to other injured tubules with nondilated morphology (Fig. 4, E and F). Next, we assessed whether injured tubule segments of nondilated and dilated morphology eventually recovered or atrophied and explored the odds of tubule atrophy by logistic regression analysis. This revealed no significant relationship between the presence of nondilated morphology and atrophy of a given tubule segment (*P* = 0.29, OR = 0.54; Fig. 4G). In contrast, tubules with dilated morphology demonstrated a fourfold increase in the odds of atrophy (*P* < 0.018, OR = 3.9; Fig. 4G), consistent with an 80% probability of atrophy. Together, these data characterize a distinct VCAM1-positive population of severely injured tubule segments, demonstrating dilated morphology, increased PDGFRβ cell recruitment, decreased NADH autofluorescence, and a higher risk of developing tubule atrophy.

**Fig. 5.**
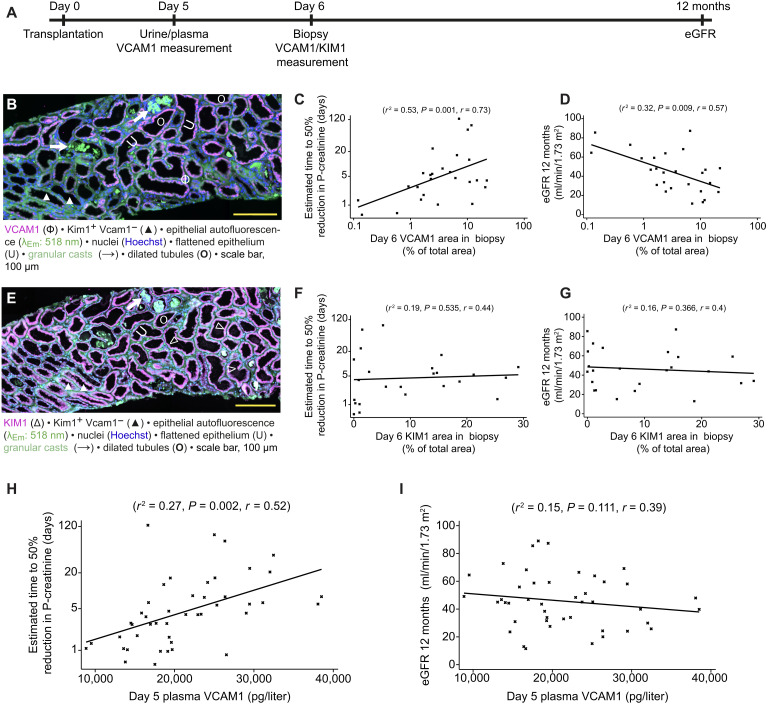
Tubular VCAM1 as a biomarker for AKI-CKD transition in humans. (**A**) Clinical study design with time points of plasma/urine/biopsy sampling for VCAM1/KIM1 measurements and eGFR assessment after human kidney transplantation. (**B**) Representative wide-field fluorescence microscopy image of a day 6 human kidney transplant biopsy stained for VCAM1 (Φ). Note the VCAM1 positivity of tubules with dilated morphology (U). (**C** and **D**) Multivariable regression analysis of patients’ tubular VCAM1 expression determined in kidney biopsies obtained 6 days posttransplantation (*n* = 21 males and 5 females) versus the estimated time to 50% reduction of plasma creatinine (C) and 12-month eGFR levels (D), respectively. *r*^2^ and Pearson’s correlation coefficient (*r*) are reported (data S1, p and q). (**E**) Representative wide-field fluorescence microscopy image of the same day 6 human kidney transplant biopsy as displayed in (B) stained for KIM1 (△). Note the abundant KIM1 expression across the sample including VCAM1-negative tubule segments (▲). (**F** and **G**) Multivariable regression analysis of patients’ tubular KIM1 expression determined in kidney biopsies obtained 6 days posttransplantation (*n* = 18 males and 6 females) versus the estimated time to 50% reduction of plasma creatinine (F) and 12-month eGFR levels (G), respectively. *r*^2^ and Pearson’s correlation coefficient (*r*) are reported (data S1, r and s). (**H** and **I**) Multivariable regression analysis of patients’ day 5 plasma VCAM1 levels posttransplantation versus the estimated time to 50% reduction of plasma creatinine (H) and 12-month eGFR levels (I), respectively. *n* = 30 males and 15 females and *n* = 29 males and 15 females for (H) and (I), respectively. *r*^2^ and Pearson’s correlation coefficient (*r*) are reported (data S1, t and u).

### VCAM1 is an early biomarker for nephron loss and AKI-CKD transition

Atrophic tubules may degrade over time resulting in atubular glomeruli (*41*–*43*), with complete replacement of tubular epithelium by scar tissue in human kidney failure biopsies (*14*). Our data indicated that day 4 VCAM1 positivity marked a tubule population with increased likelihood of atrophy (Fig. 4), while day 20 VCAM1 positivity served as a selective marker for atrophic tubules (Fig. 2, C and D). Furthermore, we also demonstrate that atrophic tubules eventually degrade (Fig. 2J and fig. S8), suggesting epithelial VCAM1 expression as an early marker of nephron loss.

Hypothesizing that nephron loss negatively affects long-term kidney function, we tested whether VCAM1 may predict AKI-CKD transition in the setting of human kidney transplantation. scRNA-seq after AKI in humans confirmed up-regulation of VCAM1 in tubules (*9*, *49*). Therefore, we analyzed VCAM1 levels in graft biopsies, plasma, and urine samples from kidney transplant recipients included in the CONTEXT trial (*50*) (Fig. 5A) and correlated this with early and 12-month graft function adjusting for recipient age and sex.

In posttransplant day 6 biopsies, immunostaining demonstrated VCAM1 positivity of tubules with dilated morphology (Fig. 5B). To determine early graft function, we estimated the time to a 50% reduction in plasma creatinine (tCr50) upon transplantation (*51*). Multivariable linear regression analysis demonstrated a significant correlation of tubular VCAM1 levels at day 6 and tCr50 (*P* = 0.001, *r*^2^ = 0.53, *n* = 26; Fig. 5C) and a negative correlation with estimated glomerular filtration rate (eGFR) at 12 months (*P* = 0.009, *r*^2^ = 0.32, *n* = 26; Fig. 5D). Thus, increased tubular VCAM1 expression at day 6 is associated with reduced early and long-term graft function after kidney transplantation.

As KIM1 is a well-established marker of human proximal tubule injury (*52*), we next examined whether tubular KIM1 expression at day 6 posttransplantation also correlated with graft function. Direct comparison of VCAM1 and KIM1 expression in the same graft biopsies revealed abundant expression of KIM1, whereas VCAM1 expression appeared more confined to a distinct subpopulation of tubules (Fig. 5, B and E, and fig. S12, A and B). Multivariable linear regression analysis demonstrated no significant correlation of tubular KIM1 levels at day 6 with early and late graft function, respectively (Fig. 5, F and G). These results indicated that some KIM1-positive tubules recovered and that nonrecovering tubule segments were more reliably identified by VCAM1 expression.

Last, we investigated whether day 5 plasma and urinary VCAM1 levels correlated with early and 12-month graft function posttransplantation. Day 5 plasma VCAM1 levels were significantly associated with tCr50 (*P* = 0.002, *r*^2^ = 027, *n* = 45; Fig. 5H) and trended toward a negative correlation with eGFR at 12 months (*P* = 0.1; Fig. 5I). In contrast, urinary VCAM1 levels were not associated with early and 12-month graft function (fig. S12, C and D).

Consistent with the notion that IRI-induced tubular atrophy is a main driver of nephron loss, these findings suggest that early tubular VCAM1 expression is associated with increased AKI and decreased 12-month kidney graft function after human kidney transplantation.

## DISCUSSION

In this study, we used serial intravital imaging of transgenic mice to track tubulointerstitial remodeling over time and decipher the spatiotemporal processes involved in the decision of successful or failed repair upon tubule necrosis. Our data reveal that atrophy development and fibrosis are associated with an early molecular switch of the tubule epithelium characterized by metabolic dysfunction, pronounced epithelial flattening, luminal dilation, and de novo expression of VCAM1. We show that tubular atrophy progresses to degradation and ultimately results in nephron loss. Consistently, we report a negative association of tubular VCAM1, but not KIM1, with acute and long-term graft function after human kidney transplantation. The impact of fibrosis on nephron fate was determined by the presence of preexisting tubular injury. Fibroblast accumulation among injured tubules was associated with an increased risk of atrophy. In contrast, fibroblast accumulation had no effect on the fate of adjacent uninjured tubules, did not induce secondary tubular injury, and appeared protective against later tubular damage.

The decision of whether tubules undergo successful repair or progress to atrophy occurs downstream of tubule necrosis (*7*). However, the mechanisms underlying this decision process remain incompletely understood (*2*–*5*) and might be orchestrated by failed tubule repair and fibrosis in the kidney. Partial IRI represents a rapid and severe model of AKI (*8*) and demonstrates features of AKI-CKD transition, including metabolic dysfunction (fig. S11), fibrosis (fig. S6), and inflammation (fig. S2). Serial imaging of partial IRI kidneys allowed a comprehensive analysis of the dynamic structural and functional changes that precede atrophy. At the same time, the presence of uninjured tissue in the same organ specifically allowed exploring of dynamic and distinct interactions of fibroblasts with injured and uninjured tissue over time and relative to tubule fate.

scRNA-seq studies have identified a population of late emerging injured tubule cells characterized by de novo expression of VCAM1 and a proinflammatory and profibrotic gene signature indicative of failed repair (*9*, *10*). Notably, the transition of proximal tubule cells toward this inflammatory phenotype is promoted by activator protein 1 and involves coexpression of ICAM1 (*48*). After IRI, VCAM1-positive cells emerge from day 2 (*10*), gradually increase in number over the following weeks (*10*, *11*) and, lastly, decrease again at late time points (*53*). Furthermore, tubule cells undergoing failed repair have been suggested to contribute to AKI-CKD transition (*10*), and tubular VCAM1 expression eventually marks atrophic tubules after IRI (*8*). However, whether recovery from this state is possible has not been explored, and little is known about structural and functional attributes of early VCAM1-expressing tubules. Here, we show abundant VCAM1 expression in entire tubule segments from day 4 after reperfusion, which was preceded by primary centers of tubule necrosis and subsequent luminal accumulation of necrotic debris resulting in progressive injury of downstream tubule segments. De novo expression of VCAM1 labeled a distinct population of severely injured tubules characterized by dilated morphology, decreased epithelial NADH autofluorescence, and high enclosure by PDGFRβ cells. The presence of this phenotype predicted atrophy development with an 80% probability, and atrophic tubules equally revealed depleted NADH autofluorescence, persistent accumulation of PDGFRβ cells, and VCAM1 positivity.

Consistent with declining VCAM1 levels at late time points after IRI (*53*), we show that atrophic tubules may eventually undergo full degradation and thereby contribute to nephron loss. Stereological analyses of different kidney disease models documented that atrophic nephrons progress toward atubular glomeruli (*41*–*43*) and biopsies from patients with kidney failure often show complete replacement of tubular epithelium with fibrotic scars (*14*). Atubular glomeruli do not contribute to glomerular filtration (*17*, *41*–*43*) and may drive hypertrophy of remnant glomeruli, progressive injury, and AKI-CKD transition according to the Brenner hypothesis (*54*). Here, we demonstrate that epithelial VCAM1, but not KIM1, is an early tissue biomarker of nephron loss and AKI-CKD transition. Using human material from a controlled clinical trial (*50*), we show that epithelial VCAM1 in day 6 biopsies correlated positively with the time to early graft recovery and negatively with 1-year graft function after kidney transplantation in humans. Consistent with these findings, tubular VCAM1 expression has also been shown to predict poor outcomes in diabetic kidney disease (*55*). In contrast, the well-established biomarker for human proximal tubule injury, KIM1 (*52*), failed to correlate with early and late graft function in our cohort. Consistent with our findings, a previous study demonstrated no significant correlation of tubular KIM1 expression and delayed graft function after human kidney transplantation (*56*). These results suggest that KIM1 expression in the absence of VCAM1 reflects a less severe and reversible state of injury, whereas VCAM1 expression denotes a specific molecular switch of the injured epithelium, which is associated with a worsened outcome.

VCAM1-positive tubule cells were spatially associated with fibrosis (*12*, *13*). Here, we show that, starting from day 2, PDGFRβ cell recruitment to atrophic tubules was consistently of higher magnitude than to recovering tubules and persisted throughout the observation period. In contrast, recovering tubules only experienced transient recruitment of PDGFRβ cells. We demonstrate that early and accelerated PDGFRβ cell recruitment was associated with increased odds of atrophy and preceded the transition from a dilated injury state toward a collapsed atrophic state. Furthermore, ECM deposition around atrophic tubules continued to increase as long as PDGFRβ cell remained present. Notably, interstitial fibrosis was suggested to constrict tubules (*17*) and disrupt kidney architecture via self-sustained and expanding ECM deposition (*5*). However, experimental evidence for these assumptions is largely lacking to the best of our knowledge.

Nevertheless, PDGFRβ cell recruitment around atrophic tubules did not further increase over time. After peaking at day 7, the abundance of PDGFRβ cells gradually declined, and their proliferation ceased. Last, we show that following laser-induced tubule atrophy and degradation, PDGFRβ cell recruitment resolved completely, indicating that fibrosis is not self-sustained. Nephrons are fragile structures lacking supportive connective tissue boundaries and may therefore depend on a stabilizing framework provided through fibrosis upon degradation of their neighbors. Thus, it is plausible that fibrosis provides a framework for surviving nephrons, which ceases upon completion of this task.

Previous studies indicated a close spatial interplay of (myo)fibroblasts with injured rather than uninjured tubules (*14*, *26*, *57*, *58*). However, the lack of longitudinal data has hampered excluding direct harmful effects of fibrosis on uninjured tissue. Whether fibrosis may cause secondary tubule injury is a longstanding question and controversially discussed (*14*, *15*). Using spatiotemporal tracking of fibroblast interactions with nonischemic and postischemic tissue in partial IRI kidneys, we demonstrate that fibrosis remained restricted to injury sites and did not infiltrate uninjured tissue over time. Nevertheless, neighboring tissue of injury sites was still exposed to higher PDGFRβ cell recruitment and fibrosis. Thus, we systematically investigated whether increased exposure of an initially uninjured tubule to PDGFRβ cells was linked to secondary tubule injury at later time points. Our findings revealed an inverse relationship between the extent of PDGFRβ cell recruitment and the likelihood of subsequent injury, suggesting that fibrosis did not induce secondary tubular damage but rather conferred a protective effect. Tubule necrosis is tightly linked to necroinflammation that may perpetuate further tissue injury and inflammation (*7*, *59*, *60*). ECM depositions around atrophic and degrading tubules might shield surrounding tissue from released danger-associated molecular patterns and cytokines. Consistent with such an assumption, ECM components were shown to prevent cytokine diffusion from tumors (*61*).

The limitations of our study include restrictions in imaging depth that prevented us from reconstructing entire nephrons. Individual tubule segments are therefore treated as independent statistical units although tubule segments belonging to the same nephron will inevitably share the same outcome and are thus not independent of each other. Furthermore, our longitudinal follow-up of partial IRI kidneys was limited to 3 weeks, as abundant tubule atrophy caused tissue shrinkage and eventually detachment of the kidney from the wAIW. This prevented documenting tubule degradation after partial IRI and sometimes caused missing data from some FOVs at late acquisition time points. For statistical data analysis, all available serial imaging was included. To accommodate an unequal number of observations per mouse, we used a linear mixed-effects model with mouse ID as random effect. No data were excluded.

In this translational study, we provide important insights into AKI-CKD progression and address the longstanding question of whether fibrosis is inherently harmful. Our findings show that VCAM1 expression in severely injured tubules marks a decisive molecular shift downstream of necrosis, preceding nephron loss and kidney dysfunction. Alongside, accelerated and persistent fibroblast recruitment around VCAM1-positive tubules preceded atrophy, yet fibrosis remained localized to injury sites and appeared protective against secondary tubular damage. These results highlight that tubular injury is a key determinant of nephron fate and advocate for a more nuanced understanding of the impact of fibrosis in kidney disease.

## MATERIALS AND METHODS

### Study approval

All experimental procedures involving animals were approved by the local authorities (Animal Experiments Inspectorate, Denmark, permit numbers: 2020-15-0201-00443 and 2024-15-0201-01839) and reported according to the Animal Research: Reporting of In Vivo Experiments guidelines. Processing of human samples and data was approved by the National Committee on Health Research Ethics (M-20100269). Informed and written content was obtained from the participants. The trial was conducted according to the Helsinki Declaration.

### Animals

A total of 60 (26 male and 34 female) mice with a mean weight of 22.3 ± 0.5 g and an age of 12.6 ± 0.6 weeks (means ± SEM) were included in the study (tables S1 and S2). Transgenic mice were obtained from the Jackson Laboratory and bred in the animal facilities at the Department of Biomedicine, Aarhus University: PDGFRβ-CreERT2–Salsa6F reporter mice were generated by crossing B6.Cg-Pdgfrbtm1.1(cre/ERT2)Csln/J (strain no. 030201) (*27*) and B6(129S4)-Gt(ROSA)26Sortm1.1(CAG-tdTomato/GCaMP6f) Mdcah/J)] (strain no. 031968) (*28*). After induction with tamoxifen, this strain identifies PDGFRβ cells by Salsa6F, a fusion protein of Genetically-Encoded Calcium Indicator-6f (GCaMP6) and tdTomato. In addition, CycB1-GFP reporter mice [Tg(Pgk1-Ccnb1/EGFP)1Aklo/J] (strain no. 023345) (*36*) were used to detect renal cell proliferation through transient nuclear/cytosolic expression of GFP expression stages S, G_2_, and M of the cell cycle.

Mice were randomly allocated to one of three groups: (i) in vivo imaging, (ii) GFR and urine collection, and (iii) ex vivo imaging, as summarized in table S2. Group i was further divided into mice undergoing partial IRI surgery, sham surgery, or laser injury, respectively. Groups ii and iii were divided into mice undergoing partial IRI surgery and sham surgery. In addition, some mice from group i were also used for ex vivo correlative microscopy upon euthanizing. Our study design precluded blinding of the experimental treatment, as the validation of surgical outcomes required close microscopic examination during the procedure. Serial intravital microscopy of partial IRI animals was challenged by strong tissue remodeling that sometimes caused missing data points from late acquisition time points. To compensate for missing data points, we included higher n numbers in the partial IRI group. For statistical analysis, all available serial imaging data were included. No data were excluded.

### Surgery

Before surgery, mice were accommodated in groups of two to five within ventilated cages (Techniplast model GM500 or GM9000) at 21° ± 2°C and 55 ± 10% relative humidity. Pups were weaned at 3 weeks of age. Mice were maintained on a standardized 12-hour light-dark diurnal cycle, with unrestricted access to standard diet and water. The bedding material consisted of Aspen wood chips measuring 2 to 3 mm in size (ABEDD, bedding midi), complemented by compressed cotton tubes for nesting purposes. Cages were enriched with Mouse Igloo with spinning wheels, wooden chew sticks, cardboard tubes, and Shepherd Shacks. Upon surgery, Mouse Igloo and cardboard tubes were replaced by cardboard box houses to prevent the abdominal image window from getting stuck. Cages were routinely cleaned once a week. To investigate whether (myo)fibroblasts engage in progressive and self-perpetuating tubulointerstitial fibrosis, two individual surgery procedures were performed. First, a unilateral partial IRI was done by occluding one branch of the left renal artery for 21 min, as previously described (*8*). Second, a wAIW was placed above the postischemic and nonischemic parts of the partial IRI kidney and implanted into the left flank of the mouse as described previously (*29*, *62*). Thus, it was possible to image the border between the ischemic and nonischemic regions.

During surgery, mice received analgesia through intraperitoneal administration of buprenorphine [0.1 mg/kg of body weight (BW); TEMGESIC] and anesthetized with isoflurane (3.5% for induction, 1.5 to 1.75% for maintenance, flow rate of 1.2 to 1.8 liters/min, and 50% oxygen in medical air). Surgery was conducted on a heating plate set to 37° ± 0.5°C, while eye ointment prevented cornea dehydration. The mouse’s left flank was shaved, and the skin was disinfected using chlorhexidine (0.5% in 70% ethanol). Hereafter, an incision into the left flank exposed the kidney. The kidney was gently repositioned, and the right artery branch was occluded with a clamp for 21 min. During occlusion, kidney temperature and hydration were maintained with a nonwoven swab and occasional flushing with 37°C saline. Upon reperfusion, a purse-string suture connected the muscle layer and the skin around the incision, and the kidney glued to the wAIW using 50 μl of cyanoacrylate glue. Last, the skin was secured around the groove of the wAIW by tightening of the purse-string suture. Sterile saline (10 μl/g of BW) was injected intraperitoneally for fluid supplementation. Following the image session at day 0, meloxicam (1 mg/kg of BW; Metacam) was administrated subcutaneously as postoperative analgesia, as well as at days 1 and 2 postsurgery. In addition, mice had restricted access to normal drinking water but had to drink water containing buprenorphine [1 ml of buprenorphine (0.3 mg/ml) to 35 ml of water] until 3 days postsurgery.

### GFR and albumin/creatinine ratio

To assess the effects of partial IRI on renal function, we monitored GFR transcutaneously over a 7-week period in freely moving mice subjected to either sham surgery (*n* = 5) or partial IRI (*n* = 8) using transcutaneous GFR monitoring (Medibeacon, Germany). Briefly, mice were anesthetized with isoflurane (3.5% induction dose) and thoroughly shaved using depilatory cream. A miniaturized fluorescence detector (Medibeacon, Germany) was then fixated to the ribs of each animal, and a fluorescein isothiocyanate (FITC)–conjugated inulin analog, sinistrin, was administered via retro-orbital injection (5 mg/100 g of BW; Medibeacon). Isoflurane was discontinued, and FITC-sinistrin clearance was recorded over a period of 90 min in awake mice. GFR was analyzed from FITC-sinistrin clearance according to the three-compartment model as outlined in the instruction manual.

Albuminuria was evaluated by measuring the albumin/creatinine ratio in spot urine samples over a 4-week period (*n* = 3 sham and *n* = 9 partial IRI). Urine samples were collected and immediately frozen at −80°C until analysis. Urinary albumin and creatinine levels were quantified using a commercially available mouse albumin enzyme-linked immunosorbent assay kit (Nordic Biosite, product no. E99-134) and a creatinine urinary detection kit (Thermo Fisher Scientific, product no. AIACUN), respectively.

### Image acquisition protocol

In vivo imaging was conducted using an upright Olympus FVMPE-RS two-photon microscope (Olympus, Japan) operated with Fluoview FV31S software (Olympus, Japan), and an upright Ultima Investigator Plus multiphoton setup (Bruker Corporation, Billerica, MA, USA) with Praire View IV software as described previously (*8*). The Olympus microscope was equipped with a MaiTaiHP DS-OL excitation laser (Spectra Physics, USA), a 25× XLPLN25xWMP2 objective, water immersion [Olympus, Japan; numerical aperture (NA), 1.05; working distance (WD), 2.00 mm], with an infrared cut filter of 690 nm, and the following detection cubes: channel 1 (Ch1), λ_Em_ = 705/45 nm [multialkali photomultiplier tube (PMT)]; Ch2, λ_Em_ = 610/35 nm (multialkali PMT); Ch3, λ_Em_ = 540/40 (GaAsP); Ch4, λ_Em_ = 480/40 (GaAsP).

The Bruker microscope was equipped with a 20× Olympus XLUMPLFLN objective, water immersion (Olympus, Japan; NA, 1.00; WD, 2.00 mm), a 720 sp. filter, and the following detection cubes: Ch1, λ_Em_ = 525/50 nm (GaAsP); Ch2, λ_Em_ = 525/50 nm (GaAsP); Ch3, λ_Em_ = 460/50 nm (GaAsP).

Before imaging, 1 μl/g of BW of a conjugated albumin–Alexa Fluor 594 dye solution (Alexa594-albumin; 2.5 mg/ml; Invitrogen) and 10 μl of PI (0.25 mg/ml; Thermo Fisher Scientific) were administered via retro-orbital injection. For imaging, the wAIW implant was slotted into a custom three-dimensional (3D) printed frame, ensuring image stabilization on an upright microscope setup (*29*). During imaging, anesthesia and temperature were maintained with a low-flow (30 to 50 ml/min, 1.0 to 1.5%) isoflurane vaporizer (SomnoSuite, Kent Scientific), which was equipped with a heating blanket placed beneath the animal.

We identified ≈4 FOV, which were imaged using dual-track excitation using 750 and 940 nm, respectively (512 × 512; dwell time, 4 μs; line averaging × 2; Galvano laser). Z-series, with a step size of 1 to 2 μm and a depth of ≈60 μm, was acquired from each FOV, starting from the capsule. Upon the end of the image session, mice recovered from anesthesia and placed back in their cage. This process was repeated for each imaging day as detailed in table S2.

To induce selective laser injury in a few nephrons within the otherwise healthy PDGFRβ-CreERT2-tdTomato kidneys (*n* = 4), we used the two-photon laser as a micromanipulator and focused high laser power on a kidney region of ~60 μm^2^. Tissue remodeling at the injury site was then monitored via serial in vivo imaging for up to 4 weeks as detailed in tables S1 and S2. At some image sessions, a tile scan approach was used if the damaged area exceeded the normal FOV. These tiles were later stitched using the cell sense software (Olympus, Japan).

After the last imaging session, mice were anesthetized with isoflurane and perfusion fixated with 4% paraformaldehyde (PFA). For correlative microscopy, the cortical renal area attached to the wAIW was separated as a ≈2-mm-thick tissue section, postfixated with 4% PFA for 1 hour, and then washed in phosphate-buffered saline (PBS) (3× for 5 min). Whereafter, they were stained ex vivo.

### Ex vivo (correlative) microscopy of VCAM1 and PDGFRβ

Kidney tissue stained against VCAM1 was blocked in 1% bovine serum albumin (BSA)/2% Sea Block/0.1% Triton X-100/PBS for 1 hour and then incubated in rabbit anti-VCAM1 (Abcam, AB0134047; 1:200) for 72 hours at 4°C. After washing in PBS (3× for 5 min), donkey anti-rabbit Alexa Fluor 405 (Jackson ImmunoResearch, 711-175-152; 1:500) secondary antibody was incubated overnight at 4°C.

For PDGFRβ, the kidney tissue was blocked with 1% BSA + 2% Sea Block in PBS for 1 hour and incubated in rabbit anti-PDGFRβ (Abcam, ab32570; 1:200) overnight at 4°C. After washing in PBS (3× for 5 min), the sample was incubated in donkey anti-rabbit Alexa Fluor 594 (Invitrogen, A21207; 1:500).

After incubation in secondary antibody, samples were washed in PBS and embedded in PBS between two coverslips separated by a 2-mm-thick spacer (SunJin Lab). Hereafter, they were imaged on an Olympus FVMPE-RS two-photon microscope. When possible, the same tissue regions as imaged in vivo were found and imaged again as described previously (*62*).

Ex vivo imaging of PDGFRβ was performed on the Olympus microscope using the same setup as described above. For ex vivo imaging of VCAM1, the following filter sets were applied: Ch1, λ_Em_ = 725/20 nm (multialkali PMT); Ch2, λ_Em_ = 570/30 nm (multialkali PMT); Ch3, λ_Em_ = 521.5/16.5 (GaAsP); Ch4, λ_Em_ = 452.5/22.5 (GaAsP). For a detailed overview, see table S3.

### Microscopy of VCAM1, PDGFRβ, KIM1, SGLT2, CD68, αSMA, PCK1, and Picrosirius Red on paraffin-embedded sections

Ex vivo samples used for paraffin embedding were postfixated in 4% PFA, whereafter they were transferred to 70% ethanol and incubated overnight. The ethanol concentration was sequentially increased to 96% for 2 hours and then to 99% for an additional 2 hours, followed by overnight incubation in 99% ethanol with xylene. Samples were subsequently embedded in paraffin at 60°C and stored until staining.

Kidney tissue was cut coronally. Upon staining, sections were dehydrated to remove water using xylene and decreasing amount of ethanol. Antigen retrieval was performed with Heat-Induced Epitope Retrieval at pH 9. Samples were blocked in 50mM NH4Cl for 30 minutes, followed by 1% BSA, 0.2% gelatine, and 0.05% saponin in PBS (pH 7.4). Primary antibodies [rabbit anti-VCAM1 (Abcam, AB0134047; 1:500) or rabbit anti-VCAM1 conjugated to Alexa Fluor 647 (Abcam, AB194319-1001; 1:200), rabbit anti-PDGFRβ (Abcam, ab32570; 1:200), rabbit anti-CD68 (Abcam, 125212; 1:900), mouse anti-αSMA (Dako, M085; 1:1000), and rabbit anti-PCK1 (Abcam, 7035; 1:200)] were diluted in 0.1% BSA and 0.3% Triton X-100 in PBS (pH 7.4) and incubated overnight at 4°C.

Samples are washed 3× for 10 min in 0.1% BSA, 0.2% gelatine, and 0.05% saponin in PBS (pH 7.4), followed by a 1-hour incubation with donkey anti-rabbit Alexa Fluor 488 (Invitrogen, A21206; 1:1000), donkey anti-rabbit Alexa Fluor 680 (Invitrogen, A10043; 1:1000), and goat anti-mouse Alexa Fluor 488 (Invitrogen, A32723; 1:1000) diluted in 0.1% BSA and 0.3% Triton X-100 in PBS (pH 7.4). After staining, samples are washed for 100 min in PBS and mounted with Glycergel (Dako, C0563) containing 2.5% (w/v) 1,4-diazabicyclo[2.2.2]octane (Merck, 803456). Samples were imaged on a Zeiss LSM900 inverted confocal laser scanning microscope with Airyscan 2 detection array. For a detailed overview, see table S4.

For costaining against KIM1 and SGLT2, sections were rehydrated using tris-buffered saline with 0.05% Tween 20 (TBST), and antigen retrieval was performed using heat and antigen unmasking solution (Vector Laboratories, H-3300-25). Subsequently, samples were blocked in TBST containing 10% donkey serum (TBST-Ser) for 30 min and then incubated in TBST-Ser with goat anti-KIM1 (R&D Systems, AF1817; 1:100) and rabbit anti-SGLT2 (Abcam, ab85626; 1:250) for 1 hour. Then, samples were washed three times for 5 min each with TBST and incubated in donkey anti-goat Alexa Fluor 647 (Jackson ImmunoResearch, 705-607-003; 1:500) and donkey anti-rabbit Rhodamine Red-X (Jackson ImmunoResearch, 711-297-003; 1:300) in TBST-Ser for 30 min. After washing three times for 5 min each in PBS, samples were incubated in 1× TrueBlack Plus (Biotium, 23014) in PBS for 5 min. Sample were quickly rinsed three times in PBS before coverslips were mounted onto the slides with ProLong Glass (Invitrogen, P36981) and sealed using clear nail polish. Samples were imaged using a Leica STELLARIS 8 inverted confocal microscope using a 10× dry objective with 3-μm *z*-step. For a detailed overview, see table S4.

Picrosirius Red staining was performed as described before (*63*): Upon deparaffinization, sections were consecutively incubated in solutions of Celestine Blue, Harris Hematoxilin, and Sirius Red. Collagen was visualized using confocal microscopy (LSM900 inverted confocal laser scanning microscope with Airyscan 2 detection array) using an excitation of 561 nm and detection of Sirius Red signal at 635 to 685 nm.

### Image analysis

During the analysis, all tubular segments from each FOV were sequentially numbered, reidentified on consecutive imaging time points, and qualitatively assessed for categorization into one of the following classifications: “undamaged,” “damaged,” “recovered,” or “atrophic.” Tubular segments were classified as damaged if they exhibited any of the following criteria: positive staining for PI, dilated or collapsed tubule lumen, luminal granular cast accumulation, flattened tubule epithelium, visually decreased NADH autofluorescence, and/or, in the case of PT-S1, visually reduced albumin endocytosis. Undamaged tubule segments were defined by the absence of all characteristics mentioned above. Tubule segments were classified as atrophic if they adopted a strongly collapsed, nonreversible state with decreased NADH autofluorescence. Recovered tubules were identified with any of the damage characteristics outlined above and later adopted an undamaged state. In addition, atrophic tubule segments were further subclassified as “degraded” if they completely disappeared. The classification of individual tubule segments across consecutive imaging time points was performed and confirmed by two experimenters.

Denoising of images was conducted using the 3-D block-matching algorithm (BM3D) (*64*), as previously described (*65*). Briefly, a denoising pipeline was created using both Fiji (*66*) and MATLAB (R2023b). Fiji facilitated batch data input/output, handled multichannel images, and merged denoised outputs. MATLAB executed BM3D algorithms, processing one channel at a time by reading temporary files written on disk by Fiji and producing the denoised data in separate temporary files.

A few of the tile scan images were shading corrected in Fiji with the BaSiC plugin by extracting the individual tiles with a script macro for each channel. The tiles were then concatenated to a single stack, and the shading correction frame was estimated in BaSiC (λ = 5.0). The shading correction image with an average intensity equal to 1 was then tiled to a grid of identical dimensions as the data and used to normalize it.

Registration of serial imaging data was performed using a combination of a manual landmark-based volumetric registration within the Fiji plugin BigWarp using rigid rotation transforms (*67*) and intensity-based medical image registration using Elastix (v5.1.0) (*68*).

To quantify PDGFRβ cell accumulation, two different approaches were used. In the first method, PDGFRβ cells were segmented using Ilastik (v1.4.0) (*34*). Here, tdTomato-labeled PDGFRβ cells were segmented from the registered 940 tracks using pixel classification. To train Ilastik, labels were assigned to z-stack images to identify structures/cells as either tdTomato-positive or -negative cells. Following exportation of the 2D Ilastik predictions, a threshold was applied using the Otsu method (*69*), whereafter the percentage of the of tdTomato cell area was assessed relative to the total FOV area (fig. S2, B and C). This approach provided an unbiased quantification of PDGFRβ cell abundance on a per-FOV basis.

In the second method, the perimeter of each individual tubule was measured for each day equivalating 6678 individual measurements. Afterward, it was measured how much of the tubule in question was in touch with surrounding PDGFRβ cells, and PDGFRβ cell enclosure was expressed as a percentage of the total perimeter of the tubule (Fig. 2E). This approach provided insights into PDGFRβ cell dynamics with individual tubules.

SHG was quantified following image segmentation using Ilastik. The software was trained to recognize SHG signals from 940 tracks. Following the export of the 2D Ilastik predictions, a threshold was applied using the Otsu method (*69*), after which the percentage of the SHG area was evaluated relative to the total FOV area. All SHG analysis was done on decapsulated, ex vivo samples to avoid bias when separating interstitial SHG signal from SHG signal deriving from the kidney capsule.

NADH signal quantification was only performed on images acquired on the Olympus system to maintain consistent detection optics for the quantification. In 750-nm excitation track data, a region of interest was manually drawn around the epithelium of each tubule, and the average intensity of channel 4 was measured.

Immunostaining and Picrosirius Red staining quantifications were performed in Fiji upon intensity thresholding. Colocalization of PDGFRβ and αSMA in ex vivo samples was determined by thresholding in respective image channels using the Otsu method (*69*). Ultimately, the colocalization between tdTomato and αSMA was determined by finding the intersection between the two processed images.

### Single-nucleus RNA-seq data analysis

We used the publicly available dataset from 10.5281/zenodo.10722341 (GSE218376, GSE151167, and GSE139107). Analyses were performed in R (version 4.3.0) using the Seurat package (v5). Cell filtering, normalization, and integration were performed as previously described (*47*). We subset only proximal tubule cells from IRI models. RunPCA, RunUMAP, and FindNeighbors functions were applied with default parameters. Unbiased clusterization was performed using FindCluster at resolution of 0.1. A low resolution was chosen, as our clusters of interest were well separated from healthy proximal tubule cells, resulting in seven clusters. Cluster annotation was performed manually, as previously described (*47*). Briefly, we used classical proximal tubule cell (PTC) markers Low-density Lipoprotein Receptor-related Protein 2 (Lrp2), Cubilin, Solute Carrier Family 5 Member 2, Solute Carrier Family 22 Member 6, Solute Carrier Family 13 Member 3, Cytochrome P450 Family 7 Subfamily B Member 1, the injury marker Hepatitis A virus cellular receptor 1 (Havcr1), and the failed repair marker Vcam1. We manually grouped all clusters with classical PTC markers and no injury or failed repair markers into the “PTC” cluster, named the Havcr1-positive and Vcam1-negative cluster “Injured,” and the failed repair marker, Vcam1-positive cluster “Failed_Repair.” DotPlot and FeaturePlot functions from Seurat (v5), as well as dittoDimPlot from the dittoSeq package, were used for data visualization. The FindAllMarkers function was used to identify top markers among clusters with a min.pct = 0.25 and a logfc.threshold = 0.25.

### Human samples

Human plasma and urine samples and kidney graft biopsies were obtained from the randomized controlled clinical trial [CONTEXT (*70*)]. The CONTEXT cohort included 225 participants undergoing kidney transplantation with a deceased donor at four European transplant centers. The participants were randomized to either perioperative remote ischemic conditioning of the leg or sham. For the present study, Danish participants with available plasma and urine sampling at day 5 after kidney transplantation, who had either anuria and need for dialysis pretransplant or a day 6 protocol biopsy, were included. Tubule, plasma, and urinary VCAM1 levels were not influenced by the remote ischemic conditioning (fig. S13). Hence, all results were pooled irrespective of intervention for further analysis in the present study.

Early graft function posttransplantation was determined by mathematical modeling of the estimated tCr50 after transplantation, as described before (*51*). Briefly, for each patient, we included all plasma creatinine measurements from the time of transplantation until 30 days posttransplant or the last posttransplant dialysis for mathematical modeling of tCr50 using an exponential, logistic, or linear model. Twelve-month graft function posttransplantation was measured as eGFR using the Chronic Kidney Disease – Epidemiology Collaboration formula (*71*) without correction for race. Human plasma and urine samples were analyzed for VCAM1 levels using the R-PLEX Mouse VCAM1 Antibody Set (Meso Scale Diagnostics, USA) according to the manufacturer’s instructions. Samples were diluted 1000 and 50 times in diluent for plasma and urine, respectively, and added in duplicates to the assay plate in a randomized order. Samples were measured on a MESO QuickPlex SQ 120 (Meso Scale Diagnostics).

Stainings of human biopsies for VCAM1 or KIM1 were done on 3-μm paraffin sections on StarFrost Adhesive object glass (Hounisen, 2510.1250). Sections were incubated at 60°C for 1 hour. To deparaffinize the sections, xylene was used, followed by hydration through a graded series of ethanol solutions: 99, 96, and 70% ethanol, each for 2 cycles of 10 min. Antigen retrieval was performed using heat-induced epitope retrieval (heating for 6 min at 700 W) with a pH 9 buffer.

The primary antibody, rabbit anti-VCAM1 (Abcam, AB0134047; 1:500 dilution) or goat anti-KIM1 (Biotechne, AF181; 1:500), was incubated overnight at 4°C. Following primary antibody incubation, sections were washed three times for 10 min each in a buffer containing 1% BSA, 0.2% gelatin, and 0.05% saponin in PBS (pH 7.4). The secondary antibody, goat anti-rabbit Cy5 (Invitrogen, A10523; 1:500 dilution) or donkey anti-goat Alexa Fluor 647 (Invitrogen, A2144; 1:1000), was applied for 2 hours at room temperature (20°C). Both primary and secondary antibodies were diluted in a solution of 0.1% BSA and 0.3% Triton X-100 in PBS (pH 7.4). For nuclear staining, Hoechst 33342 (1 μg/ml; Invitrogen, H3570) was incubated concurrently with the secondary antibody. Sections were mounted with Glycergel mounting medium (Dako, C0563) containing 1,4-diazabicyclo[2.2.2]octane (0.025 g/ml; 2.5%, w/v; Merck, 803456). Sections without primary antibody were used as negative control. For detailed overview, see table S5.

Following staining, the biopsies were imaged on an Olympus VS120 upright wide-field fluorescence microscope (Olympus, Japan) equipped with a 20 × UPLSAPO objective (air immersion) and a high-sensitivity monochrome camera with 82% quantum efficiency (Hamamatsu, ORCA-FLASH4.0 V2). Images were captured using emission wavelengths of 518 nm (exposure time, 500 ms), 455 nm (exposure time, 100 ms), and 565 nm (exposure time, 800 ms).

For quantification of tubular VCAM1 and KIM1 expression in human kidney graft biopsies, the entire biopsy was scanned, and positive areas were quantified via thresholding of the respective channel using a set cutoff value. As glomeruli in healthy kidneys contain VCAM1 (*72*), tissue areas containing glomeruli were excluded before thresholding. To minimize bias, the quantification of VCAM1 in biopsies, plasma, and urine was conducted in a blinded manner without knowledge of respective eGFR levels of the patients.

### Statistics

Statistical analyses were performed using MATLAB version 2023b (The MathWorks Inc.). For imaging data analysis, individual tubular segments were treated as statistical units, with repeated measurements of the same tubules taken over time. To accommodate missing data points and varying sample sizes across different mice, linear mixed-effects models were constructed for each dataset obtained from segment analyses. Detailed specifications of response variables are provided in the “Legends for data S1” section in the Supplementary Materials for each specific analysis. All linear mixed-effects models accounted for individual animal subjects as random effects.

For mice imaged during the first week, data were analyzed strictly according to the specific imaging days. However, for adequate n values, in weeks 2 and 3, mice imaged on certain days were pooled with those imaged the day before or after. For example, data from day 20 include mice imaged on both day 20 and day 21.

Results from linear regression analyses are presented as the *P* value and *R*^2^ goodness of fit and effect size, which is estimated as Pearson’s correlation coefficient. Significant results from clinical data were adjusted for recipient age and sex using multivariable linear regression. Results from binary regressions are presented as *P* values and ORs. For both linear and binary regressions, the line equations (*y* = slope * *x* + intercept) are reported in data S1.

GFR and albumin-to-creatinine ratios were analyzed using paired two-way analysis of variance (ANOVA) in GraphPad Prism, with time-matched comparisons across different days for the same subjects, and Bonferroni correction was applied for multiple comparisons. Quantitative figures and related statistics were generated using GraphPad Prism, Stata, and the Gramm toolbox (*73*). Data are generally presented as means ± SEM and plotted as mean with 95% confidence intervals (CIs) in the figures.

### Reproducibility

In vivo imaging experiments of individual experimental mice were performed as independent experiments. Upon reassurance that inclusion criteria were met (successful partial IRI surgery and correct placement of the abdominal imaging window implant), FOVs were reproducibly identifiable on the basis of distinct necrotic cell damage patterns on day 0. Representative images were selected from data obtained from at least three independent experiments.

Analysis of the albumin-to-creatinine ratio from urine samples was performed as duplicates. Baseline transcutaneous GFR measurements were performed as duplicates. After IRI/sham surgery, transcutaneous GFR measurements were performed longitudinally and were hence obtained as single values per time point and animal.

### Use of artificial intelligence

Portions of this manuscript were refined with use of OpenAI’s ChatGPT (GPT-4 and GPT-5). Prompts used included “Please refine” and “Please shorten.” All artificial intelligence–assisted edits were critically reviewed, revised, and approved by the authors.
