# Supplementary material for: In vivo expression of VCAM1 precedes nephron loss following kidney tubular necrosis
Source: Sci Adv. 2025 Oct 22;11(43):eadz5358. doi: 10.1126/sciadv.adz5358 (PMC12542942; doi:10.1126/sciadv.adz5358)
Supplement: Supplementary file 1 — Figs. S1 to S13 Tables S1 to S5 Legend for data S1 [file sciadv.adz5358_sm.pdf]

Science Advances
AAAS

Supplementary Materials for

In vivo expression of VCAM1 precedes nephron loss following kidney tubular necrosis

Anders M. Kristensen *et al.*

Corresponding author: Ina Maria Schiessl, ina.maria.schiessl@biomed.au.dk;
Anders M. Kristensen, amk@biomed.au.dk

Sci. Adv. **11**, eadz5358 (2025)
DOI: 10.1126/sciadv.adz5358

The PDF file includes:

Figs. S1 to S13
Tables S1 to S5
Legend for data S1

Other Supplementary Material for this manuscript includes the following:

Data S1

Fig. S1.

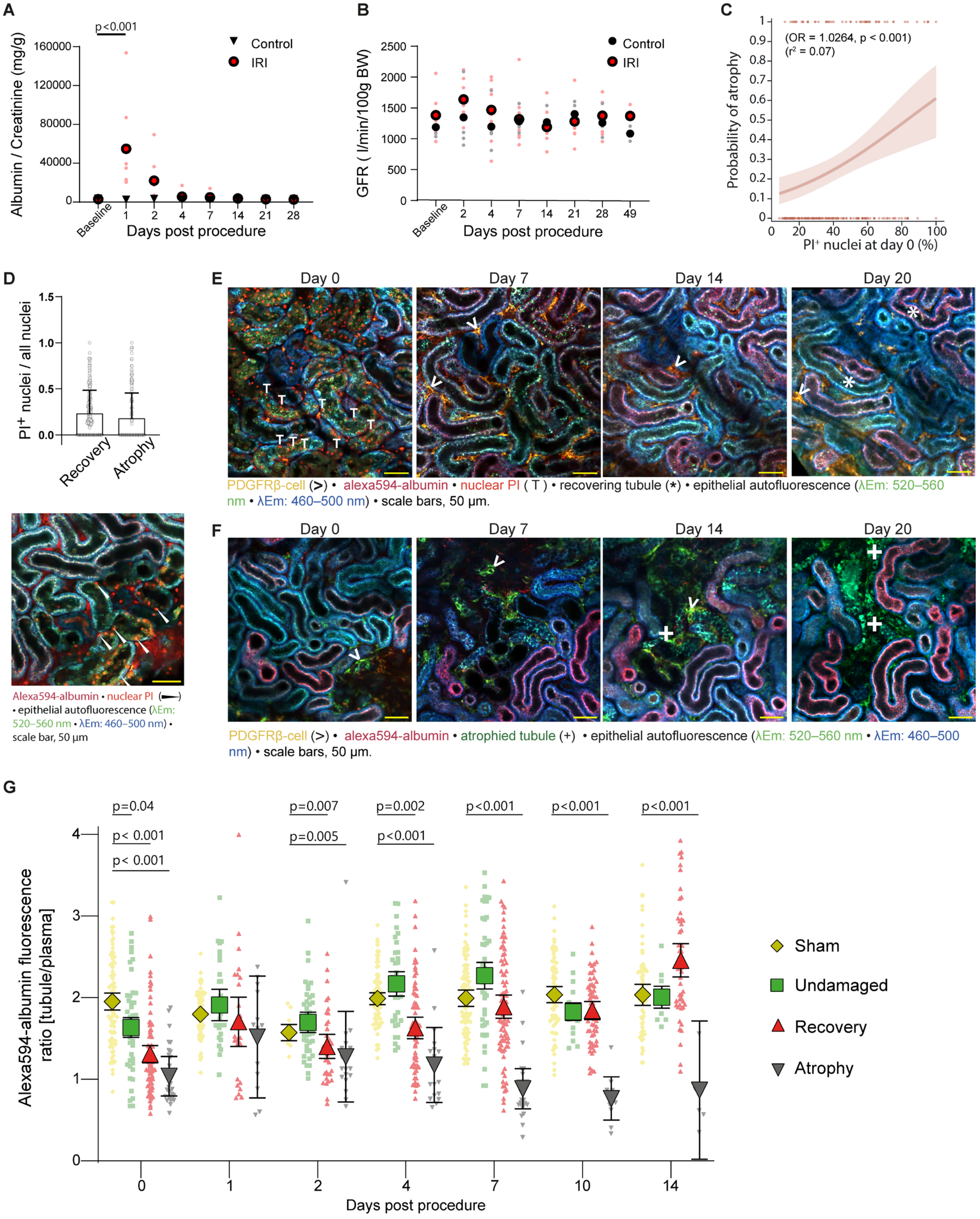

Supplementary Figure 1: Albuminuria and GFR after partial IRI.

(**A**) Albumin/creatinine ratio from spot urine collected from 2 male and 1 female sham and 5 male and 4 female partial IRI mice. Mean ± with scatterplot. Statistical test: repeated measurement two-

way ANOVA, factors: treatment and days from treatment, post-hoc analysis: multiple comparisons, Bonferroni correction (Data S1.v). (**B**) Glomerular filtration rate measured transcutaneously in awake mice including 3 male and 2 female sham and 4 male and 4 female partial IRI mice. Mean ± with scatterplot. Statistical test: repeated measurement two-way ANOVA, factors: treatment and days from treatment, post-hoc analysis: multiple comparisons, Bonferroni correction (Data S1.w). (**C**) Binary regression analysis of tubule fate (0 = non-atrophy, 1 = atrophy) and the percent of nuclei positive for Propidium Iodide (PI) per tubule segment (n = 512 tubules from 5 male and 5 female mice). Line equation, r2 and effect size as odds ratio (OR) for the predictor are reported (Data S1.x). (**D**) Quantification of PI+ nuclei (in % of all nuclei per segment) in tubules which recover (n = 235) and atrophy (n = 147) from 5 male and 5 female mice. Mean ± 95% CI with scatterplot. Statistical test: linear mixed-effect model, p-values from two-sided tests, (Data S1.y). (**E**, **F)** Serial in vivo 2-photon microscopy of a PDGFRβ-tdTomato reporter mouse kidney on days 0, 7, 14, and 20 post partial IRI. (**G**) Assessment of albumin reuptake over time in sham (n = 97), undamaged (n = 41), recovering (n = 86) and atrophic S1 proximal tubule segments (n = 32) from 2 male and 1 female sham and 5 male and 5 female partial IRI mice. Mean ± 95% CI with scatterplot. Statistical test: linear mixed-effect model, p-values from two-sided tests indicate differences between groups (Data S1.z).

Fig. S2.

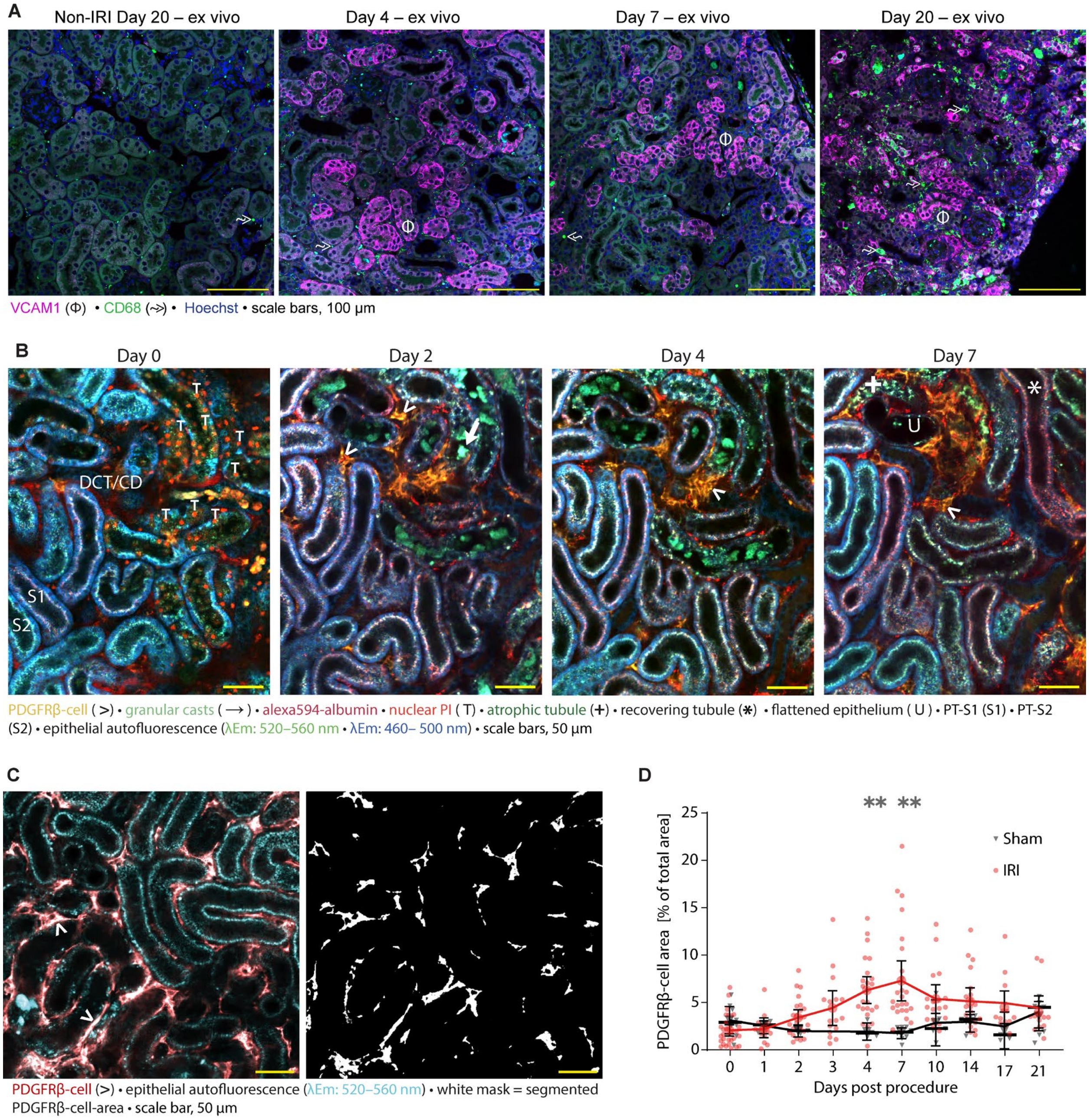

Supplementary Figure 2: Dynamic PDGFRβ-cell recruitment upon partial IRI

(**A**) Immunostaining against VCAM1 (magenta) and CD68 (green) on partial IRI kidneys harvested on days 4, 7, and 20 indicate increased macrophage infiltration in the post-ischemic tissue over time. (**B**) Serial in vivo 2-photon microscopy of a PDGFRβ-tdTomato reporter mouse kidney on days 0, 2, 4, and 7 post partial IRI, reveals tubular and interstitial remodeling prior to tubule recovery (*) and atrophy (+). (**C**) Image segmentation of PDGFRβ-cells using machine learning. (**D**) Quantification of segmented PDGFRβ-cells in sham (8 fields of view from 2 male and 1 female mice) and partial IRI kidneys (31 FOVs from 5 male and 5 female mice) over time. Mean ± 95% CI with scatterplot. Statistical test: linear mixed-effect model, p-values from two-

sided tests indicate significant difference between groups at respective days (Data S1.aa).

Fig. S3.

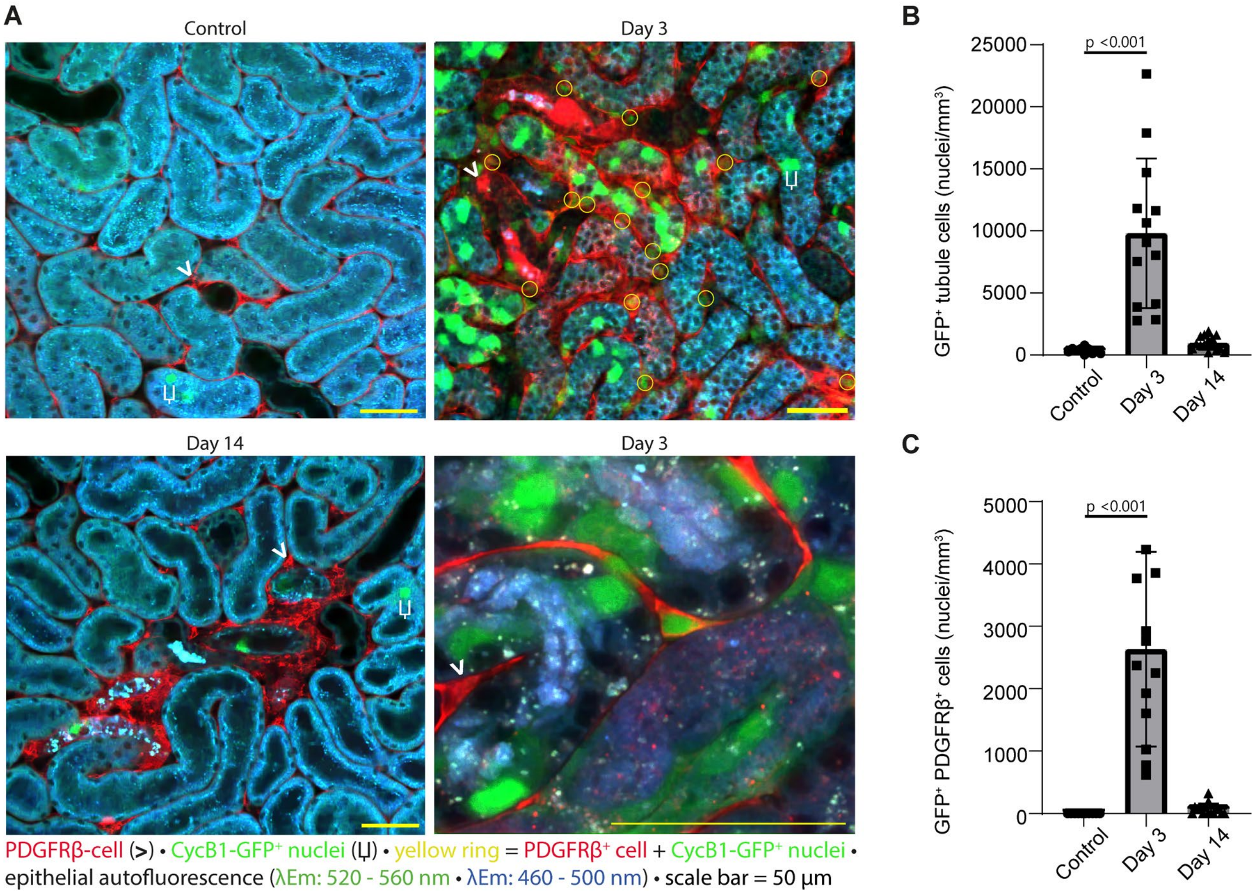

Supplementary Figure 3: PDGFRβ-cell proliferation

(**A**) Ex vivo 2-photon microscopy of CycB1-GFP reporter mouse kidneys after control and 3 days, and 14 days after partial IRI indicate proliferating cells by transient GFP-expression (green). An additional day-3 image demonstrates the GFP-expression in a proliferating PDGFRβ-cell. (**B**) Quantification of proliferating tubule cells in field of views (FOVs) from control (n = 10 FOVs from 1 male and 6 female mice) and at days 3 (n = 13 FOVs from 1 male and 3 female mice) and 14 (n = 15 FOVs from 1 male and 3 female mice) post partial IRI. Mean ± 95% CI with scatterplot. Statistical test: linear mixed-effect model, p-values from two-sided tests indicate differences between groups (Data S1.bb). (**C**) Quantification of proliferating PDGFRβ-cells in FOVs from control (n = 10 FOVs from 1 male and 6 female mice) and at days 3 (n = 13 FOVs from 1 male and 3 female mice) and 14 (n = 15 FOVs from 1 male and 3 female mice) post partial IRI. Mean ± 95% CI with scatterplot. Statistical test: linear mixed-effect model, p-values from two-sided tests indicate differences between groups (Data S1.cc).

Fig. S4.

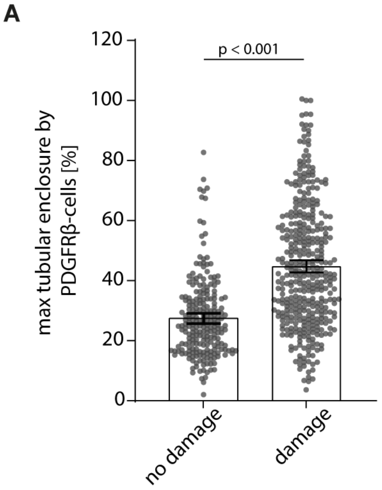

Supplementary Figure 4: Quantification of maximum tubular enclosure

(**A**) Quantification of max tubular enclosure by PDGFRβ-cells of non-damaged (n = 229) and damaged (n = 388) tubule segments from 5 male and 5 female partial IRI mice. Mean ± 95% CI with scatterplot. Statistical test: linear mixed-effect model, p-value from two-sided tests indicate significant differences between groups (Data S1.dd).

Fig. S5.

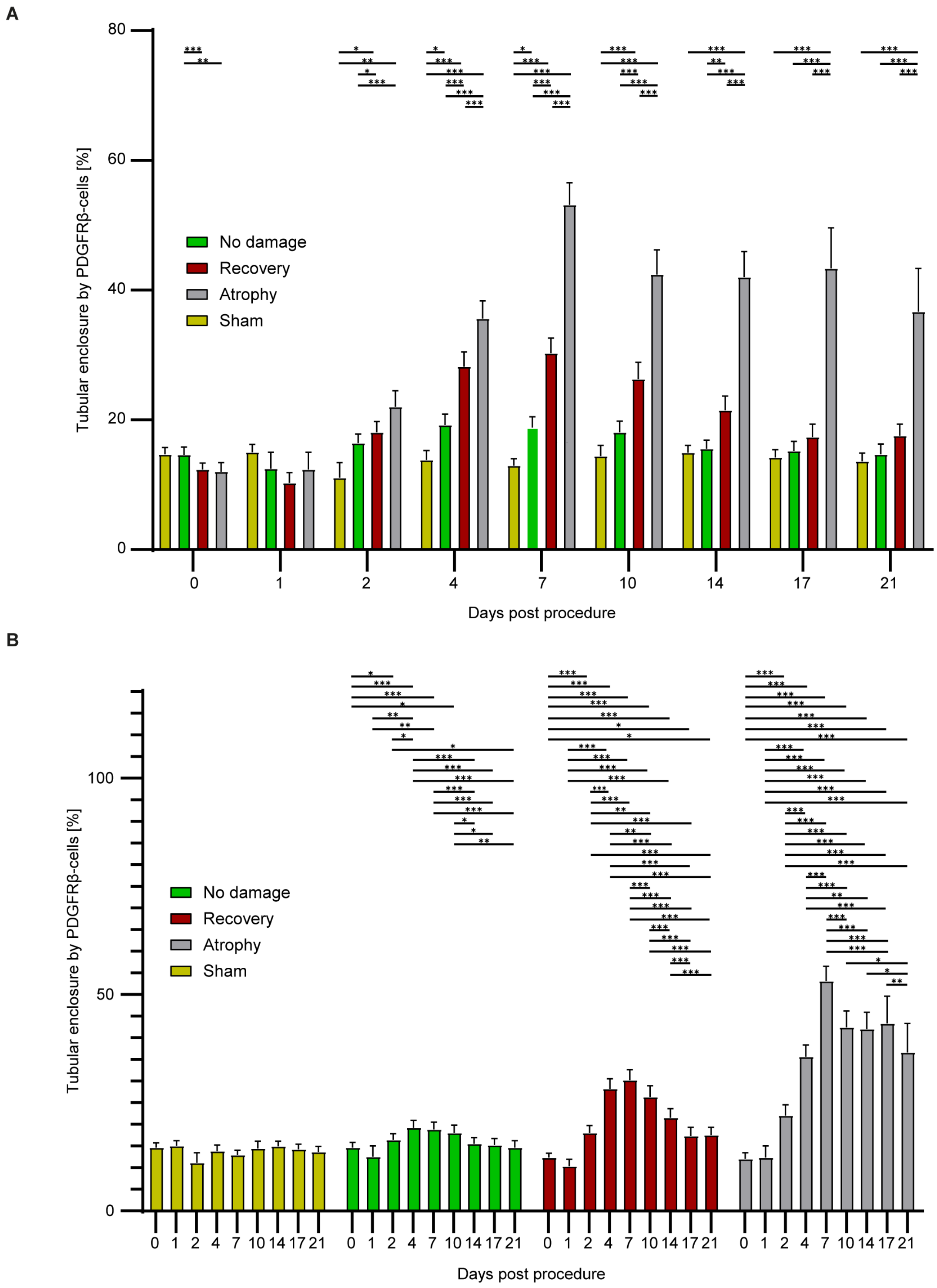

Supplementary Figure 5: Quantification of tubular enclosure by PDGFRβ-cells

Quantification of tubular enclosure by PDGFRβ-cells in tubule segments from sham mice (n = 250 tubule segments from 2 male and 1 female mice), and partial IRI mice (n = 5 male and 5 female

mice), categorized based on outcomes: no damage ($n = 225$), recovery ($n = 233$), and atrophy ($n = 142$). Data are shown as mean ± 95% CI. Statistical test: linear mixed-effect model (Data S1 .ee). *: significant difference between groups when compared at the same day (a) and between days within the same group (b); *: $p < 0.05$; **: $p<0.01$; ***: $p<0.001$.

Fig. S6.

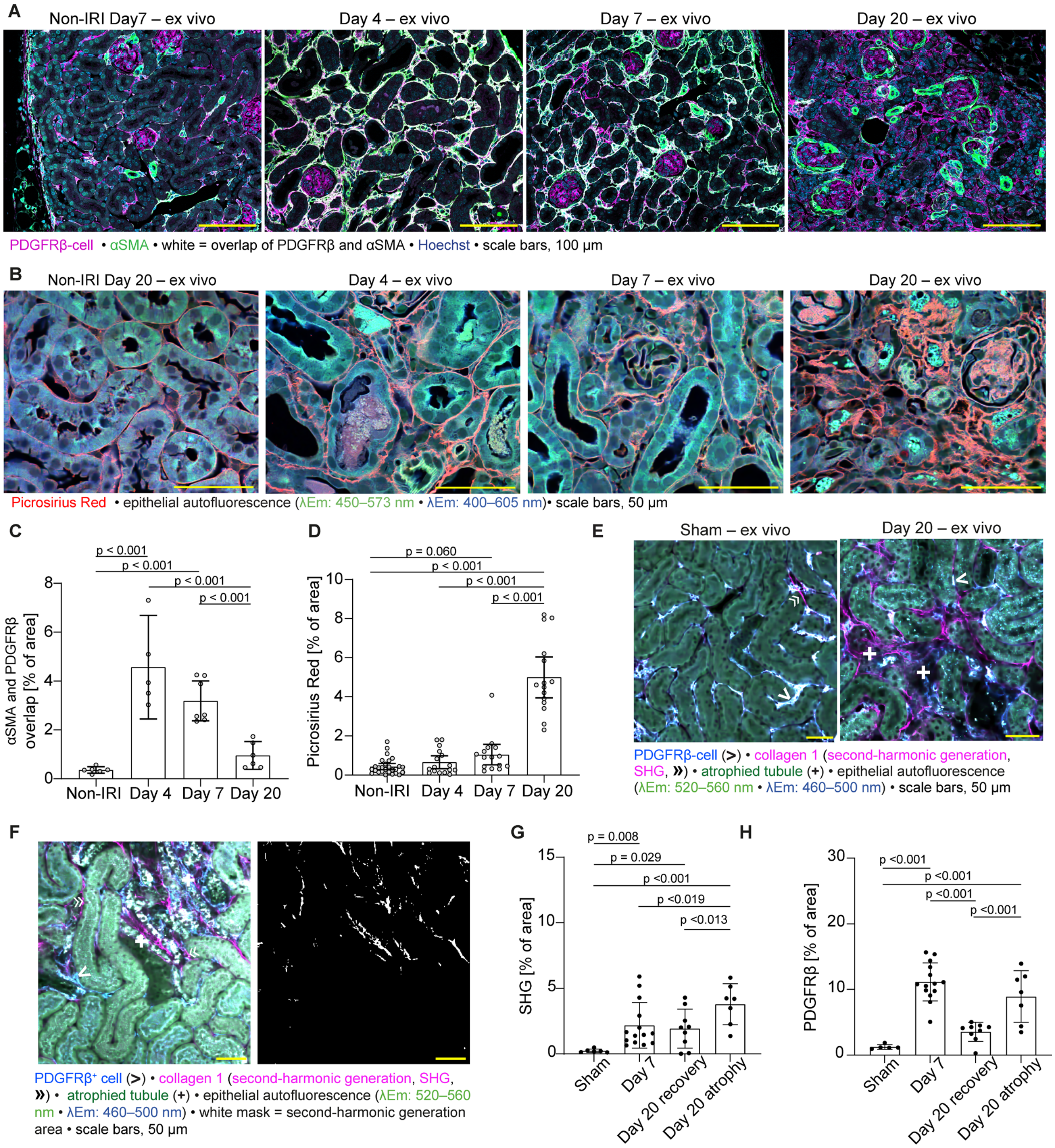

Supplementary Figure 6: Quantification of fibrosis in remodeling, recovering and atrophic regions in partial IRI kidneys

(**A**) Immunostaining against PDGFRβ (magenta) and αSMA (green) on partial IRI kidneys harvested on days 4, 7, and 20 indicate myofibroblast differentiation in the post-ischemic tissue. (**B**) Picrosirius Red staining on partial IRI kidneys harvested on days 4, 7, and 20 indicate fibrosis in the post-ischemic tissue. (**C**) Quantification of co-localization of PDGFRβ and αSMA in percent

of the total area. Each area was made as 3*3 field of views tile scan from non-IRI areas (n = 6 from 3 male and 3 female mice), day 4 (n = 7 from 2 male and 2 female mice), day 7 (n = 6 from 1 male and 2 female mice) and day 21 from (n = 6 from 2 male and 1 female mice). Data are shown as mean ± 95% CI. Statistical test: linear mixed-effect model (Data S1. ff). (**D**) Quantification of Picrosirius Red-positive area in percent of the total field of view (n = 27 from 4 male and 4 female mice), day 4 (n = 16 from 2 male and 2 female mice), day 7 (n = 15 from 1 male and 2 female mice) and day 21 from (n = 15 from 2 male and 1 female mice). Data are shown as mean ± 95% CI. Statistical test: linear mixed-effect model (Data S1.gg). (**E**) Ex vivo 2-photon microscopy of PDGFRβ-tdTomato reporter mouse kidneys after sham and 20 days after partial IRI. Collagen 1 is indicated by second harmonic generation signal (SHG, magenta). Note low SHG signal in sham and strong SHG signal in association with atrophic tubules (+, day 20) after partial IRI. (**F**) Image segmentation of SHG signal using machine learning. Quantification of the segmented SHG- (**G**) and PDGFRβ-area (**H**) in percent of the total area of regions of interest defining either sham (n = 6 from 2 male and 1 female mice), remodeling tubules at day 7 (n = 14 from 2 male and 2 female mice), and recovering (n = 9 from 1 male and 2 female mice), or atrophic tubule segments (n = 7 from 1 male and 2 female mice), at day 21 after partial IRI. Data are shown as mean ± 95% CI. Statistical test: linear mixed-effect model (Data S1.hh and ii).

Fig. S7.

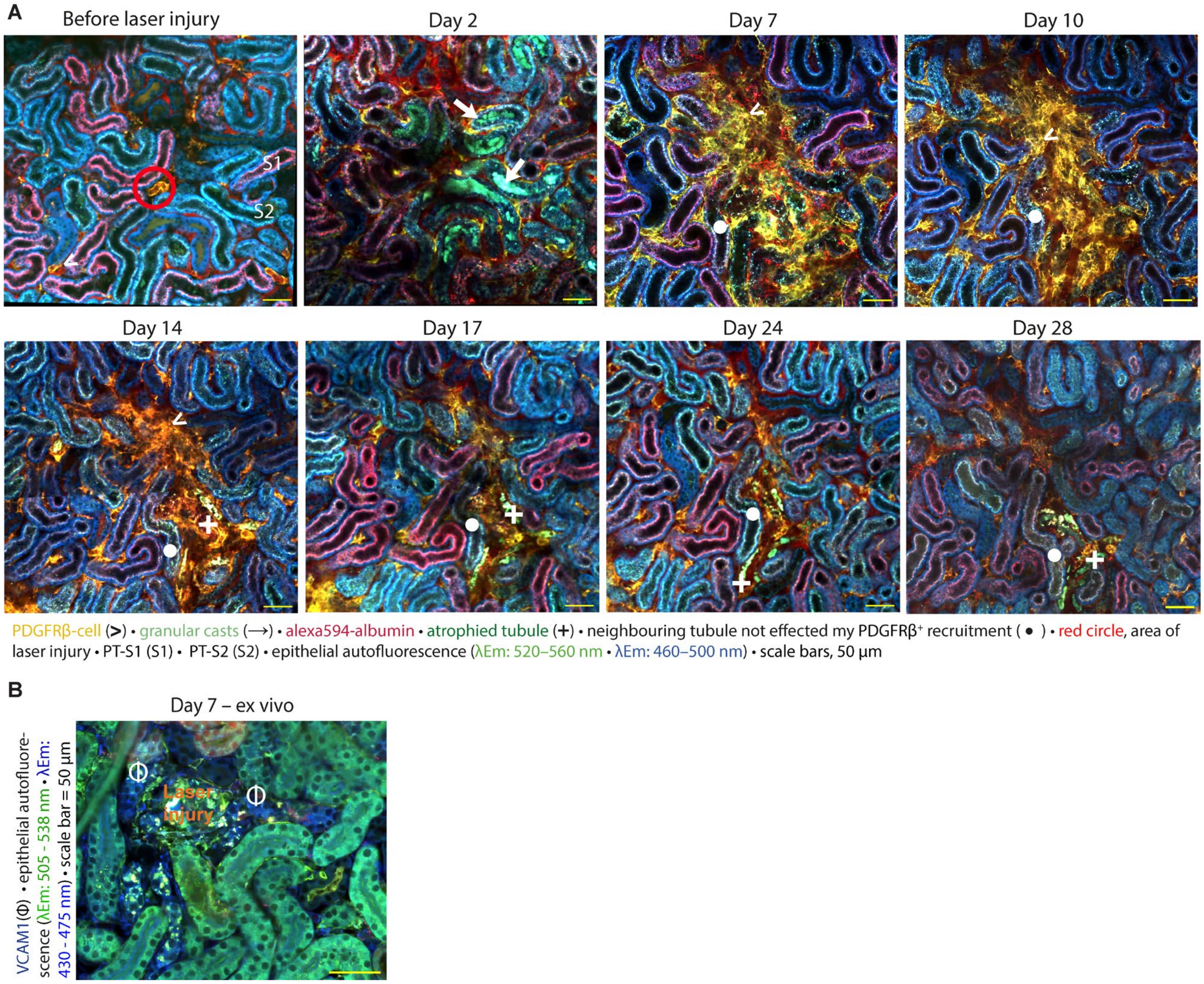

Supplementary Figure 7: Tubule remodeling after laser injury

(**A**) Serial in vivo 2-photon microscopy of a healthy PDGFRβ-tdTomato reporter mouse kidney before and days 0, 2, 7, 10, 14, 17, 24, and 28 after focal laser injury through focused 2-photon laser exposure on indicated tissue region (red circle). Focal laser injury-mediated damage eventually results in tubule atrophy (+). PDGFRβ-cell recruitment in the wound peaks at days 7 and 10 and thereafter gradually resolves with progressing wound closure. (**B**) Ex vivo 2-photon microscopy reveals VCAM1-expression (Φ, blue) in injured tubules at day 7 following laser injury.

Fig. S8.

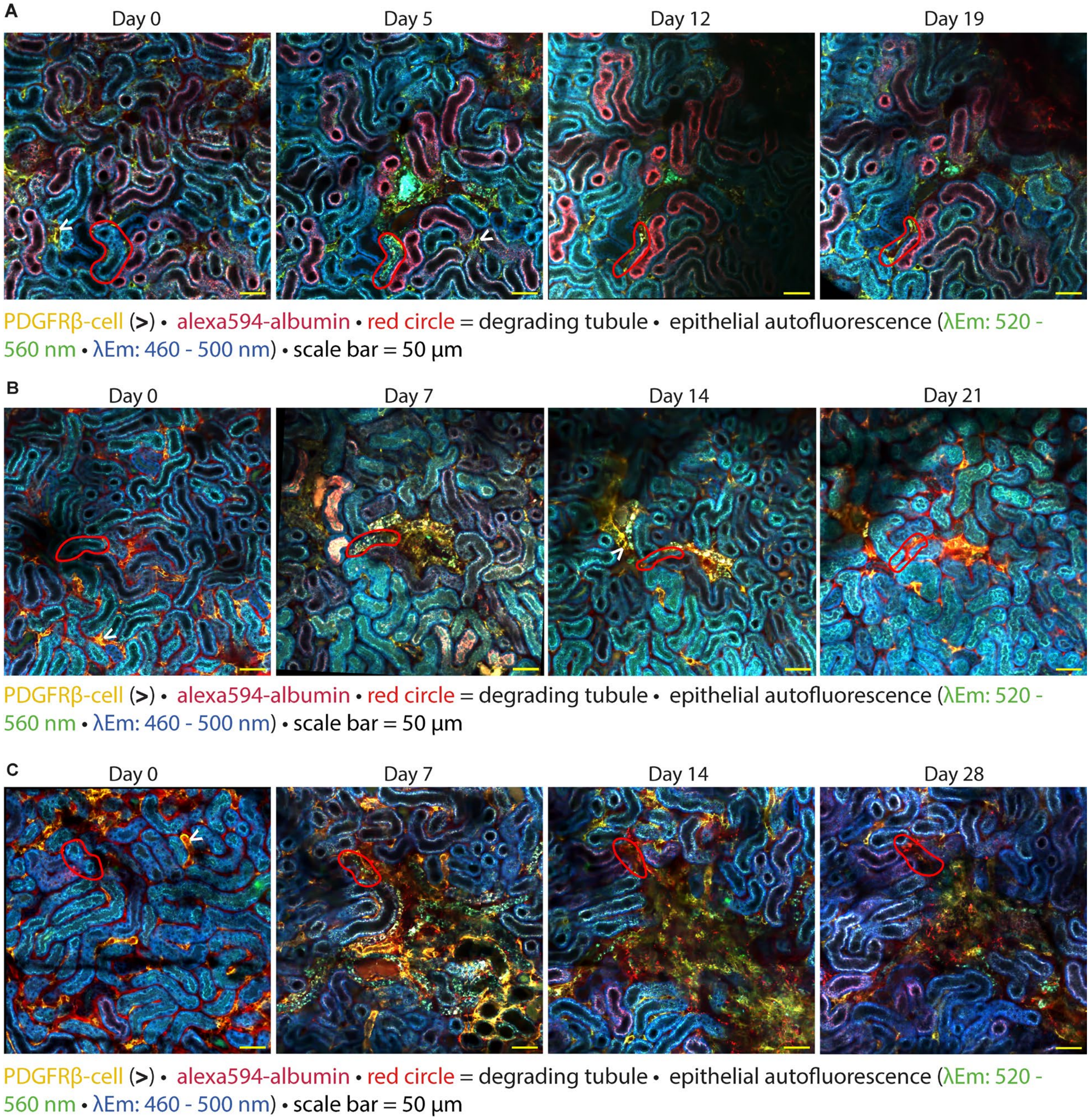

Supplementary Figure 8: Tubule degradation after laser injury

Serial in vivo 2-photon microscopy of a healthy PDGFRβ-tdTomato reporter mouse kidney imaged before and indicated days after laser injury. Red circles identify degrading tubules.

Fig. S9.

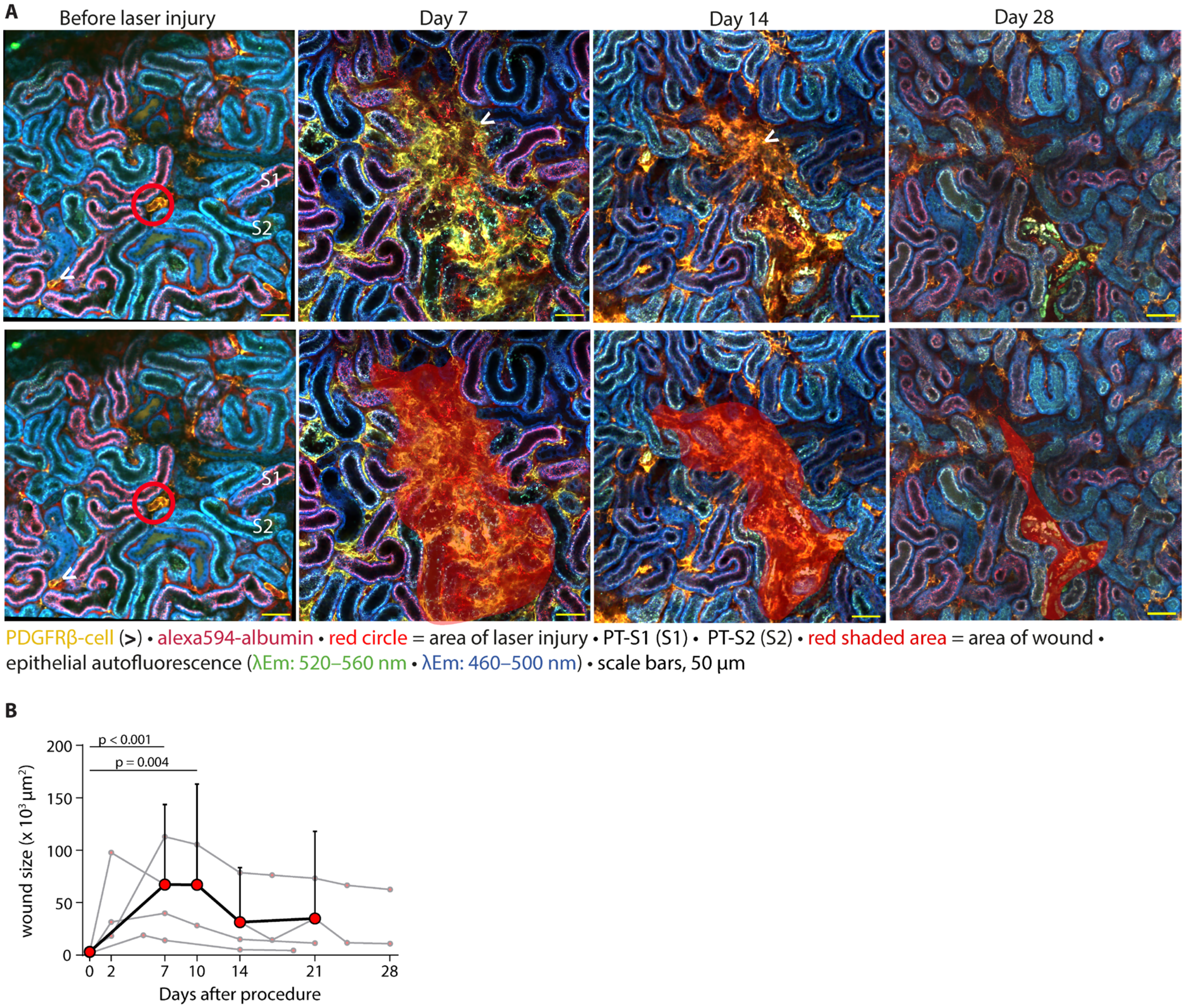

Supplementary Figure 9: Wound closure after laser injury

(**A**) Serial in vivo 2-photon microscopy of a healthy PDGFRβ-tdTomato reporter mouse kidney before and days 7, 14, and 28 after focal laser injury through focused 2-photon laser exposure on indicated tissue region (red circle) without (upper row) and with (lower row) schematic indication of the would size area (red shading). (**B**) Quantification of wound size area following focal laser injury over time (n = 4 FOVs from 1 male and 3 female mice). Mean with scatterplot. Statistical test: linear mixed-effect model, p-values from two-sided tests indicate significant difference from baseline (Data S1.jj).

Fig. S10.

A

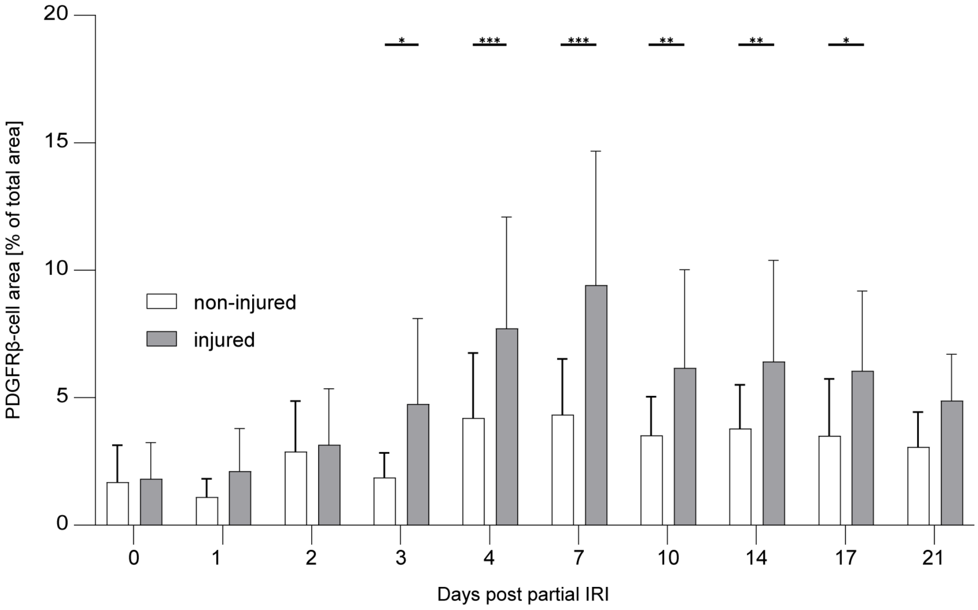

B

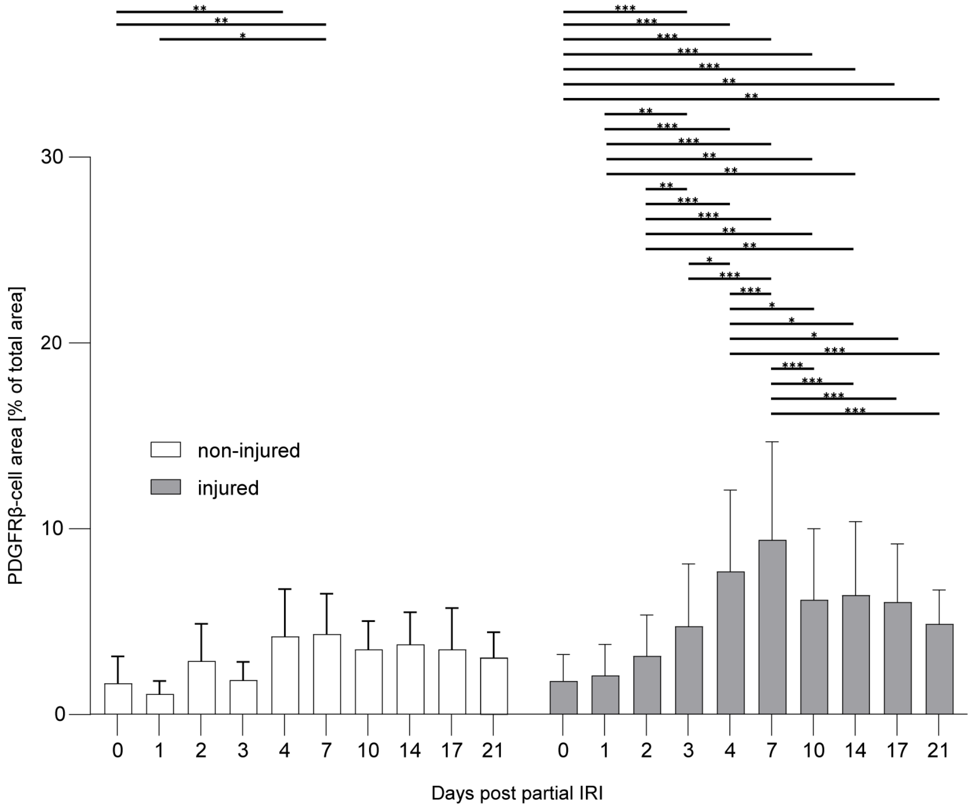

Supplementary Figure 10: Quantification of PDGFRβ-cells in non-injured and injured areas

Quantification of segmented PDGFRβ-cells in spatially distinct areas of non-injured ($n = 20$) and injured tubule segments ($n = 38$) from 5 male and 5 female mice as categorized based on serial imaging data as exemplified in Figure 3A-B. Data are shown as mean ± 95% CI. Statistical test: linear mixed-effect model (Data S1.kk). *: significant difference between groups when compared at the same day (a) and between days within the same group (b); *: $p < 0.05$; **: $p<0.01$; ***: $p<0.001$.

Fig. S11.

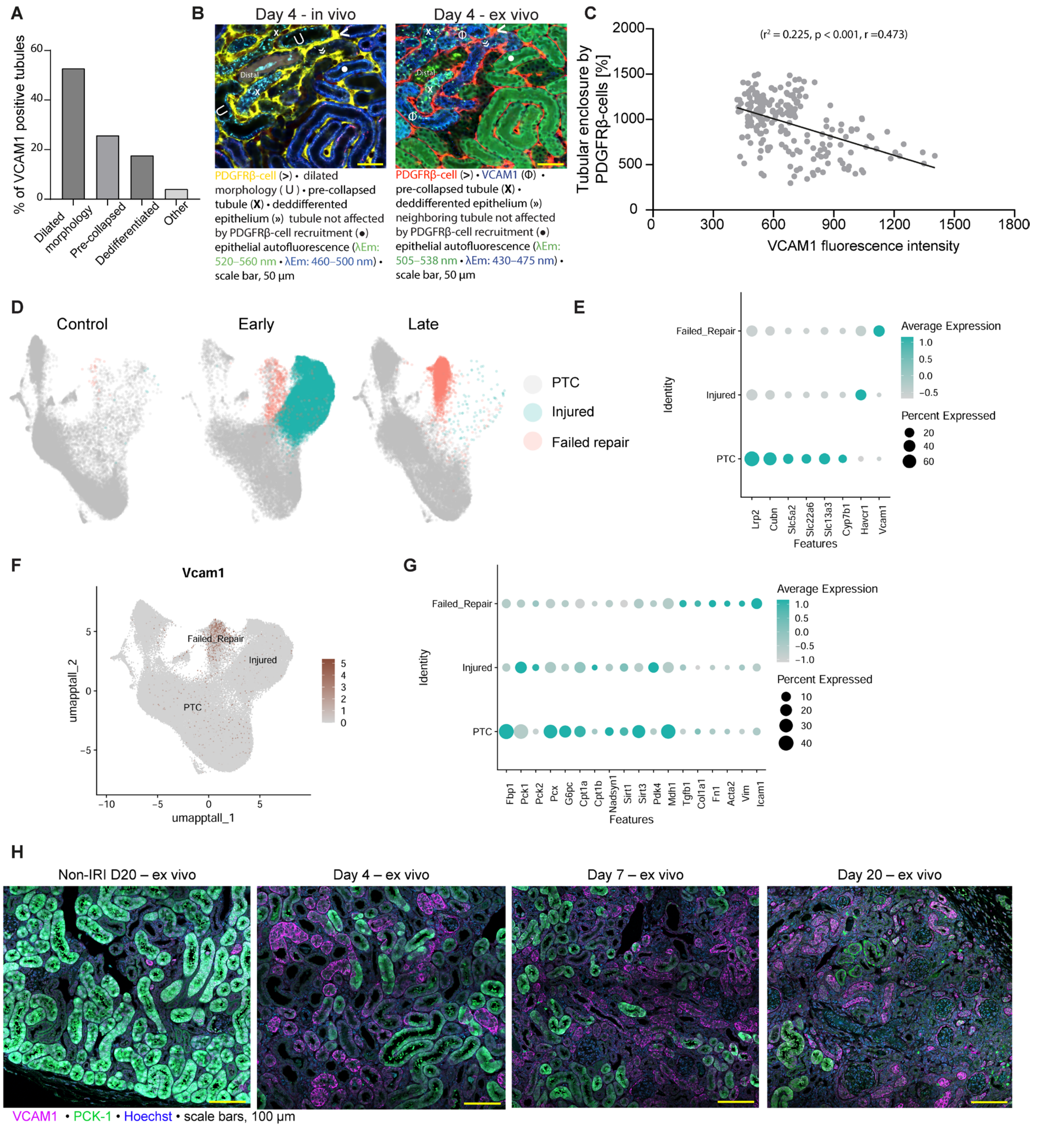

Supplementary Figure 11: Tubular VCAM1-positivity in day 4 partial IRI kidneys

(**A**) Distribution of VCAM1-positivity among tubule segments of different morphological features (n = 74 tubule segments from 3 female mice). (**B**) Representative correlative in vivo and ex vivo 2-photon microscopy of VCAM1-positive tubule segments of different morphology. (**C**) Linear regression analysis of day 4 tubular NADH autofluorescence in vivo measurements versus secondary antibody intensity after ex vivo VCAM1 immunostaining (n = 209 tubule segments from 3 female mice). r2 and Pearson's correlation coefficient (r) are reported (Data

S1.ll). (**D**) UMAP plot of single-nucleus RNAseq data of unilateral IRI mice (controls: sham-operated; early: 4 to 96 h post renal artery clamping; late: 2 and 6 weeks post renal clamping), showing major states of proximal tubule cells (PTC): normal PTC, Injured and Failed_Repair (n=79,656 nuclei). (**E**) DotPlot showing expression of markers for differentiation (Lrp2, Cubn, Slc5a2, Slc22a6, Slc13a3, Cyp7b1), injury (Kim1) and failed repair (Vcam1) across the identified cell clusters. (**F**) FeaturePlot displaying predominant expression of Vcam1 in the Failed_Repair cluster. (**G**) DotPlot showing expression of selected genes related to cellular metabolism (Fbp1, Pck1, Pck2, Pcx, G6pc, Cpt1a, Cpt1b, Nadsyn1, Sirt1, Sirt3, Pdk4, Mdh1) and pro-fibrotic pathways (Tgfb1, Col1a1, Fn1, Acta2, Vim, Icam1). (**H**) Immunostaining against VCAM1 (magenta) and PCK1 (green) on partial IRI kidneys harvested on days 4, 7, and 20 indicate a decreased PCK1-expression in VCAM1-positive tubules.

Fig. S12.

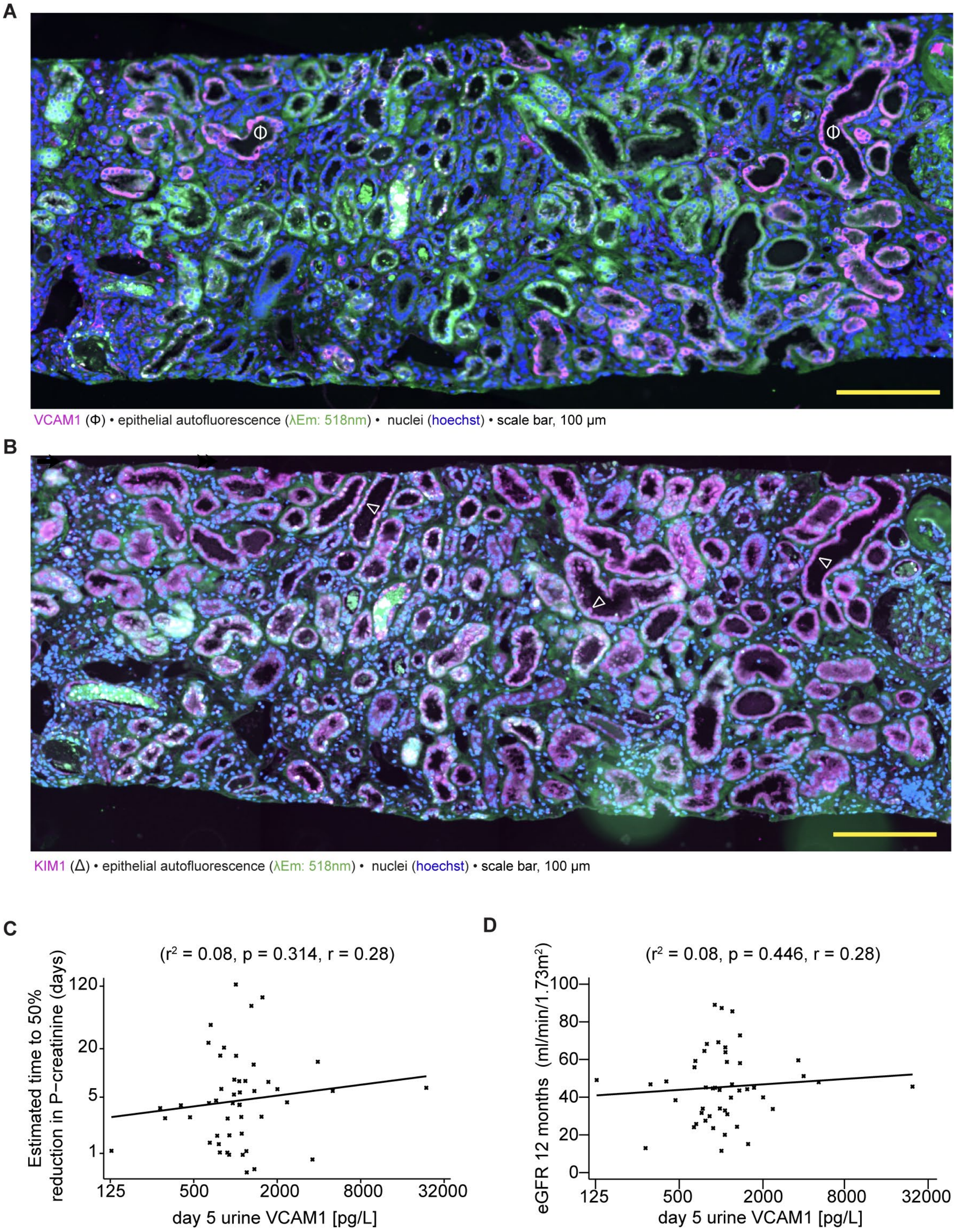

Supplementary Figure 12: VCAM1-levels in human urine and graft function

(**A-B**) Representative widefield fluorescence microscopy images of the same day 6 human kidney transplant biopsy stained for VCAM1 (Φ, **A**) and KIM1 (△, **B**), respectively. (**C**) Multivariable

regression analysis of patients' day 5 urinary VCAM1 levels post transplantation versus the estimated time to 50% reduction of plasma creatinine and 12-month eGFR levels (**D**), respectively. n = 32 males/16 females and n = 31 males/16 females for (**C**) and (**D**), respectively. r^2 and Pearson's correlation coefficient (r) are reported (Data S1. mm and nn).

Fig. S13.

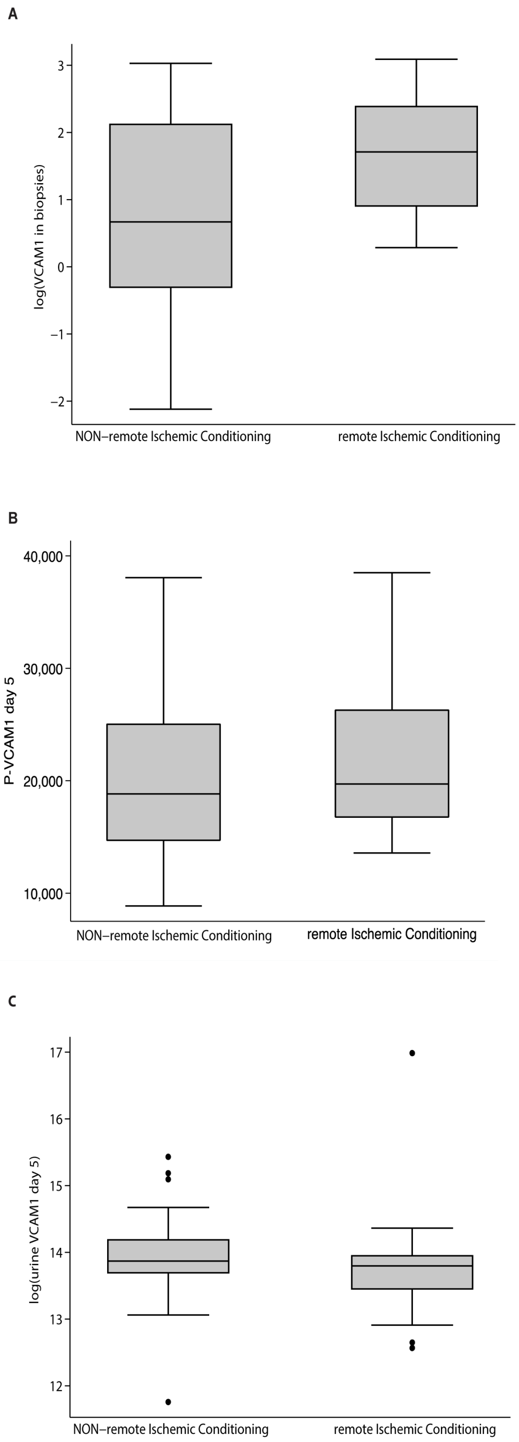

Supplementary Figure 13: No effect of remote ischemic conditioning on VCAM1 levels in tissue, plasma and urine.

(**A**) Boxplot of Vcam1 in day 6 graft biopsies of transplanted patients (n= 21 males and 5 females) that were subjected to remote ischemic conditioning (n = 12) or no remote ischemic conditioning

(n = 14). Two-sample t-test (Data S1.oo) (**B**) Boxplot of Vcam1 in day 5 plasma of transplanted patients (n= 30 males and 15 females) that were subjected to remote ischemic conditioning (n = 22) or no remote ischemic conditioning (n = 23). Two-sample t-test (Data S1.pp). (**C**) Boxplot of Vcam1 in day 5 urine of transplanted patients (n= 32 males and 16 females) that were subjected to remote ischemic conditioning (n = 22) or no remote ischemic conditioning (n = 22). Two-sample t-test (Data S1.qq).

Table S1.

Treatment	Partial IRI			Laser Injury	Sham		
**Protocol**	IVM – n = 5 (m) 5 (f)		GFR – n = 4(m) 4(f)	n = 1(m) 3(f)	IVM – n = 2(m) 1(f)		GFR – n = 3(m) 2(f)
	Unique mice	Unique segments	Unique mice	Unique mice	Unique mice	Unique segments	Unique mice
**Day 0**	10	748	8	4	3	250	5
**Day 1**	4	285	0	0	1	201	5
**Day 2**	8	618	8	2	2	35	5
**Day 3**	5	311	0	0	0	0	5
**Day 4**	6	596	8	1	2	247	5
**Day 5**	0	0	0	1	0	0	0
**Day 7**	9	671	8	3	3	271	5
**Day 8**	0	0	0	1	0	0	0
**Day 10**	6	461	0	3	2	226	5
**Day 12**	0	0	0	1	0	0	0
**Day 13**	1	22	0	0	0	0	0
**Day 14**	7	489	8	4	2	214	5
**Day 17**	4	317	0	2	2	202	5
**Day 19**	0	0	0	1	0	0	0
**Day 20**	1	91	0	1	0	0	0
**Day 21**	2	210	8	2	3	232	5
**Day 28**	0	0	8	2	0	0	5
**Day 49**	0	0	4	0	0	0	3

Supplementary Table 1: Study design.
m: male. f: female. IVM: intravital microscopy. GFR: Glomerular filtration rate

Table S2.

i) In vivo imaging							
**mouse ID**	Intervention	Gender	age (whole weeks)	Weight (g)	genotype	Days of imaging	Ex vivo staining
**#001**	partial IRI	f	21	21,4	PDGFRβ-Salsa6F	0,1,2,3,7	
**#002**	partial IRI	m	19	19,1	PDGFRβ-Salsa6F	0,3,7,14	
**#003**	partial IRI	m	17	31	PDGFRβ-Salsa6F	0,1,2,3	
**#004**	partial IRI	m	10	18,9	PDGFRβ-Salsa6F	0,1,2,3,4,7,14	
**#005**	partial IRI	f	12	23	PDGFRβ-Salsa6F	0,4,7,10,14,17,20	
**#006**	partial IRI	f	10	22,2	PDGFRβ-Salsa6F	0,2,7,10,14	
**#007**	partial IRI	f	14	24	PDGFRβ-Salsa6F	0,2,4,7,10,13,17,21	
**#008**	partial IRI	m	21	29,4	PDGFRβ-Salsa6F	0.2.4.7.10.14.17.21	Vcam1
**#009**	partial IRI	f	14	21,9	PDGFRβ-Salsa6F	0,2,4,7,10,14,18	Vcam1
**#010**	partial IRI	m	9	23,7	PDGFRβ-Salsa6F	0,1,2,3,4,7,10,14	Vcam1
**#011**	partial IRI	f	8	18,5	PDGFRβ-Salsa6F	0,2,4	Vcam1
**#012**	partial IRI	f	8	17,35	PDGFRβ-Salsa6F	0,2,4	Vcam1
**#013**	partial IRI	f	7	17,23	PDGFRβ-Salsa6F	0,2,4	Vcam1
**#014**	Sham	m	10	27,8	PDGFRβ-Salsa6F	0,2,7,14,21	
**#015**	Sham	m	12	23,7	PDGFRβ-Salsa6F	0.1.4.7.10.14.17.21	
**#016**	Sham	f	9	19,7	PDGFRβ-Salsa6F	0,2,4,7,10,14,17,21	
**#017**	Laser burn	f	11	19	PDGFRβ-Salsa6F	0,2,7,10,14,17,21,24,28	
**#018**	Laser burn	f	10	18	PDGFRβ-Salsa6F	0,2,7,10,14,17,21,24,28	
**#019**	Laser burn	f	8	19,7	PDGFRβ-Salsa6F	0,5,8,12,14,19	
**#020**	Laser burn	m	6	15,2	PDGFRβ-Salsa6F	0,7,10,14,21	

ii) GFR/urine collection

**mouse ID**	Intervention	Gender	age (whole weeks)	weight		Days of GFR	Urine
**#021**	partial IRI	m	19	28,3	PDGFRβ-Salsa6F	0,2,4,7,14,21,28,49	x
**#022**	partial IRI	m	17	32,14	PDGFRβ-Salsa6F	0,2,4,7,14,21,28,49	x
**#023**	partial IRI	f	14	19,2	PDGFRβ-Salsa6F	0,2,4,7,14,21,28,49	x
**#024**	partial IRI	f	14	20	PDGFRβ-Salsa6F	0,2,4,7,14,21,28,49	x
**#025**	partial IRI	f	16	21	PDGFRβ-Salsa6F	0,2,4,7,14,21,28	
**#026**	partial IRI	f	16	22,5	PDGFRβ-Salsa6F	0,2,4,7,14,21,28	
**#027**	partial IRI	m	16	25,6	PDGFRβ-Salsa6F	0,2,4,7,14,21,28	
**#028**	partial IRI	m	16	24	PDGFRβ-Salsa6F	0,2,4,7,14,21,28	
**#029**	Sham	m	16	24	PDGFRβ-Salsa6F	0,2,4,7,14,21,28	
**#030**	Sham	m	16	26,7	PDGFRβ-Salsa6F	0,2,4,7,14,21,28	
**#031**	Sham	m	12	31,4	PDGFRβ-Salsa6F	0,2,4,7,14,21,28,49	x
**#032**	Sham	f	11	24,2	PDGFRβ-Salsa6F	0,2,4,7,14,21,28,49	x
**#033**	Sham	f	14	17,7	PDGFRβ-Salsa6F	0,2,4,7,14,21,28,49	x
**#034**	partial IRI	m	23	27,6	PDGFRβ-Salsa6F		x
**#035**	partial IRI	f	22	21,6	PDGFRβ-Salsa6F		x
**#036**	partial IRI	f	20	20,3	PDGFRβ-Salsa6F		x
**#037**	partial IRI	m	20	28,3	PDGFRβ-Salsa6F		x
**#038**	partial IRI	m	20	31,5	PDGFRβ-Salsa6F		x

iii) ex vivo imaging

**mouse ID**	Intervention	Gender	age (whole weeks)	weight (g)		Day of image, post partial IRI	Ex vivo staining
**#039**	partial IRI	f	7	21,3	CycB1	3	PDGFRβ r
**#040**	partial IRI	m	7	20,1	CycB1	3	PDGFRβ r
**#041**	partial IRI	f	8	18,1	CycB1	3	PDGFRβ r
**#042**	partial IRI	f	8	18,8	CycB1	3	PDGFRβ r
**#043**	partial IRI	f	9	19,2	CycB1	14	PDGFRβ r
**#044**	partial IRI	f	9	21,5	CycB1	14	PDGFRβ r
**#045**	partial IRI	f	6	18,9	CycB1	14	PDGFRβ r
**#046**	partial IRI	f	9	19,2	CycB1	14	PDGFRβ r
**#047**	partial IRI	f	12	17,4	Bl6	2	KIM1/SGLT2
**#048**	partial IRI	f	12	20,9	Bl6	2	KIM1/SGLT2
**#049**	partial IRI	m	7	17,7	Bl6	2	KIM1/SGLT2
**#050**	partial IRI	m	10	24,6	Bl6	2	KIM1/SGLT2
**#051**	partial IRI	f	13	20.4	PDGFRβ-Salsa6F	21	Vcam1, CD68, PCK1, αSMA, Picosirius red
**#052**	partial IRI	m	13	28.9	PDGFRβ-Salsa6F	21	Vcam1, CD68, PCK1, αSMA, Picosirius red
**#053**	partial IRI	m	13	29.3	PDGFRβ-Salsa6F	21	Vcam1, CD68, PCK1 αSMA, Picosirius red
**#054**	partial IRI	f	11	21.3	PDGFRβ-Salsa6F	7	Vcam1, CD68, PCK1, αSMA, Picosirius red
**#055**	partial IRI	f	11	20.8	PDGFRβ-Salsa6F	7	Vcam1, CD68, PCK1,

							αSMA, Picosirius red
**#056**	partial IRI	m	11	26.8	PDGFRβ-Salsa6F	7	Vcam1, CD68, PCK1, αSMA, Picosirius red
**#057**	partial IRI	f	10	23,1	PDGFRβ-Salsa6F	4	Vcam1, CD68, PCK1, αSMA, Picosirius red
**#058**	partial IRI	f	10	22,3	PDGFRβ-Salsa6F	4	Vcam1, CD68, PCK1, αSMA, Picosirius red
**#059**	partial IRI	m	10	25,4	PDGFRβ-Salsa6F	4	Vcam1, CD68, PCK1, αSMA, Picosirius red
**#060**	partial IRI	m	10	24,5	PDGFRβ-Salsa6F	4	Vcam1, CD68, PCK1, αSMA, Picosirius red

Supplementary Table 2: Individual mice. The use of each individual mouse in the study m: male. f: female.

Table S3.

Staining for	Block	Primary antibody	Washout	Secondary antibody
**VCAM1**	1% BSA/2% SEA BLOCK/0,1 % Triton X-100/PBS for 1 hour	Rabbit anti-VCAM-1 (Abcam AB0134047, 1:200) for 72 hours at 4 °C	3 x 5 min in PBS	Donkey anti-Rabbit Alexa 405 (Jackson ImmunoResearch 711-175-152, 1:500), overnight at 4°C
**PDGFRβ**	1% BSA + 2 % SEA in PBS for 1 hour	Rabbit anti-PDGFrβ (Abcam ab32570, 1:200) overnight at 4°C	3 x 5 min in PBS	Donkey anti-Rabbit Alexa 594 (Invitrogen A21207, 1:500), room temperature for 4 hours

Supplementary Table 3: Ex vivo (correlative) microscopy

After incubation in secondary antibody, samples were washed in PBS and embedded in PBS between two coverslips separated by a 2 mm thick spacer (SunJim Lab) and imaged on an Olympus FVMPE-RS 2-photon microscope. When possible, the same tissue regions as imaged in vivo were found and imaged again.

Table S4.

Staining for	Block	Primary antibody	Washout	Secondary antibody
**KIM1**	10% donkey serum (TBST-Ser) for 30 min	Goat anti-KIM-1 (1:100, R&D Systems, AF1817) for 1 hour	3 x 5 min in PBS	donkey anti-goat Alexa Fluor 647 (1:500, Jackson ImmunoResearch, 705-607-003) for 30 min
**SGLT2**	10% donkey serum (TBST-Ser) for 30 min	Rabbit anti-SGLT2 (1:250, Abcam, ab85626) for 1 hour	3 x 5 min in PBS	donkey anti-rabbit Rhodamine Red-X (1:300, Jackson ImmunoResearch, 711-297-003) for 30 min
**PDGFRβ**	50 mM NH_4Cl in 0.01M PBS for 30 min.<br>1% BSA, 0.2% gelatin, 0.05% Saponin in 0.01M PBS for 3 x 10 min	Rabbit anti-PDGFrβ (Abcam ab32570, 1:200) overnight at 4°C	3 x 10 min in 0.1% BSA, 0.2% gelatin, 0.05% Saponin in PBS	donkey anti-rabbit Alexa 680 (1:1000, Invitrogen A10043) 1 h at 21°C
**VCAM1**	50 mM NH_4Cl in 0.01M PBS for 30 min.<br>1% BSA, 0.2% gelatin, 0.05% Saponin in 0.01M PBS for 3 x 10 min	Rabbit anti-VCAM-1 (Abcam AB0134047, 1:500) overnight at 4°C	3 x 10 min in 0.1% BSA, 0.2% gelatin, 0.05% Saponin in PBS	donkey anti-rabbit Alexa 488 (1:1000, Invotrogen A21206) 1 h at 21°C
	50 mM NH_4Cl in 0.01M PBS for 30 min.<br>1% BSA, 0.2% gelatin, 0.05% Saponin in 0.01M PBS for 3 x 10 min	Rabbit anti VCAM-1-Alexa 647 (Abcam AB194319-1001, 1:200) overnight at 4°C	3 x 10 min in 0.1% BSA, 0.2% gelatin, 0.05% Saponin in PBS	N/A
**CD68**	50 mM NH_4Cl in 0.01M PBS for 30 min.<br>1% BSA, 0.2% gelatin, 0.05% Saponin in 0.01M PBS for 3 x 10 min	Donkey anti Rabbit Alexa 488 (Invotrogen A21206, 1:1000), overnight at 4°C	3 x 10 min in 0.1% BSA, 0.2% gelatin, 0.05% Saponin in PBS	donkey anti-rabbit Alexa 488 (1:1000, Invotrogen A21206) 1 h at 21°C
**αSMA**	50 mM NH_4Cl in 0.01M PBS for 30 min.	Mouse anti αSMA (Dako M085, 1:1000), overnight at 4°C	3 x 10 min in 0.1% BSA, 0.2% gelatin,	Goat anti Mouse Alexa 488 (Invitrogen A32723, 1:1000) 1 h at 21°C

	1% BSA, 0.2% gelatin, 0.05% Saponin in 0.01M PBS for 3 x 10 min		0.05% Saponin in PBS	
**PCK1**	50 mM NH_4Cl in 0.01M PBS for 30 min.<br>1% BSA, 0.2% gelatin, 0.05% Saponin in 0.01M PBS for 3 x 10 min	Rabbit anti-PCK1(Abcam 7035, 1:200), overnight at 4°C	3 x 10 min in 0.1% BSA, 0.2% gelatin, 0.05% Saponin in PBS	donkey anti-rabbit Alexa 488 (1:1000, Invotrogen A21206) 1 h at 21°C

Supplementary Table 4: Microscopy on paraffin embedded sections

Table S5.

	Block	Primary antibody	Washout	Secondary
**VCAM1**	50 mM NH4Cl in PBS (pH 7.4) for 30 minutes, followed by three 10-minute washes in a blocking buffer containing 1% BSA, 0.2% gelatin, and 0.05% saponin in PBS (pH 7.4)	Rabbit anti-VCAM1 (Abcam AB0134047, 1:500 dilution), was incubated overnight at 4°C. the antibody was diluted in a solution of 0.1% BSA, 0.3% Triton X-100, in PBS (pH 7.4)	Following primary antibody incubation, sections were washed three times for 10 minutes each in a buffer containing 1% BSA, 0.2% gelatin, and 0.05% saponin in PBS (pH 7.4)	Goat anti-rabbit Cy5 (Invitrogen A10523, 1:500 dilution), was applied for 2 hours at room temperature (20°C). The antibody was diluted in a solution of 0.1% BSA, 0.3% Triton X-100, in PBS (pH 7.4).
**Kim1**	50 mM NH4Cl in PBS (pH 7.4) for 30 minutes, followed by three 10-minute washes in a blocking buffer containing 1% BSA, 0.2% gelatin, and 0.05% saponin in PBS (pH 7.4)	Goat anti KIM1 (Biotechne AF181, 1:500 dilution), was incubated overnight at 4°C. the antibody was diluted in a solution of 0.1% BSA, 0.3% Triton X-100, in PBS (pH 7.4)	Following primary antibody incubation, sections were washed three times for 10 minutes each in a buffer containing 1% BSA, 0.2% gelatin, and 0.05% saponin in PBS (pH 7.4)	Donkey anti Goat Alexa 647 (Invitrogen A2144, 1:1000) was applied for 2 hours at room temperature (20°C). The antibody was diluted in a solution of 0.1% BSA, 0.3% Triton X-100, in PBS (pH 7.4).
**Hoechst**				Hoechst 33342 (1 µg/mL, Invitrogen H3570) was incubated concurrently with the secondary antibody

Supplementary Table 5: Staining of human biopsies

Sections were mounted with Glysergel mounting medium (Dako C0563) containing 0.025 g/mL 1,4-diazabicyclo[2.2.2]octane (2.5% w/v, Merck 803456). Sections without primary antibodies were used as negative controls.

Data S1.

Extended_Statistics

Detailed information for the statistical tests used, references with letter to the specific statistical tests and models reported in the manuscript's figure legends. All p values reported were obtained from two-sided test.

a. Fig. 1D

Mean ± 95% CI with scatterplots. n = 275, 168, 237, 173, 164, 173, 89 for day 0, 1, 2, 3, 4, 7, and 10 respectively, from 5 male and 5 female partial IRI mice. Statistical test: linear mixed-effect model, response: luminal cast (%), fixed effect: day from partial IRI, ($F (6, 1270) = 69.684$, $p < .001$). Random effect: mouse ID.

b. Fig. 2B

Mean ± 95% CI with scatterplots. n = 266 for sham from 2 male and 1 female sham mice and n = 116 for atrophy from 3 male and 4 female partial IRI mice. Statistical test: linear mixed-effect model, response: epithelial NADH, fixed effect: group, ($F (3, 531) = 36.532$, $p < .001$). Random effect: mouse ID.

c. Fig. 2F

Mean ± 95% CI with scatterplot. n = 250 tubule segments from 2 male and 1 female mice sham mice. n = 225, 233, 142 tubule segments with no damage, recovery and atrophy respectively from 5 male and 5 female partial IRI mice. Statistical test: linear mixed-effect model, response: tubular enclosure by PDGFRβ-cells (%), fixed effect: day ($F (9, 4083) =123.13$, $p < .001$), group ($F (2, 4083) = 108.59$, $p < .001$), day/group ($F (18, 4083) = 37{,}562$, $p < .001$). Random effect: mouse ID.

d. Fig. 2G

Mean ± 95% CI with scatterplot. n = 62 tubule segments positive for VCAM1 and n = 40 negative for VCAM1 from 2 male and 1 female mice. Statistical test: linear mixed-effect model, response: tubular enclosure by PDGFRβ-cells (%), fixed effect: group, ($F (1, 100) = 86.875$, $p < .001$). Random effect: mouse ID.

e. Fig. 2I

Quantification of collagen 1 based on segmented second harmonic generation (SHG) signal and PDGFRβ-cell signal from sham (n = 6, 2 male and 1 female mice), day 7 remodeling (n = 14 from 2 male and 2 female mice), and day 20 recovered (n = 9) and atrophic areas (n = 7) after partial IRI (n = 1 male and 2 female mice). Statistical test: linear mixed-effect model, response: SHG area (% of total area), fixed effect: group ($F (3, 32) = 6{,}92$, $p = 0.001$). Random effect: mouse ID.

Quantification of segmented PDGFRβ signal from sham (n = 6, from 4 mice), day 7 remodeling (n = 14 from 3 mice), and day 20 recovered (n = 9) and atrophic areas (n = 7) after partial IRI (n = 3 mice).
Statistical test: linear mixed-effect model, response: PDGFRβ-cell area (% of total area), fixed effect: group ($F (3, 31) = 28.495$, $p < .001$). Random effect: mouse ID.

f. Fig. 2L

Mean ± 95% CI with scatterplot. (n = 3 field of views from 2 male and 1 female mice) and laser injury FOVs (n = 4 from 1 male and 3 female mice).
Statistical test: linear mixed-effect model, response PDGFRβ-cell area (% of total area), fixed effect: day, ($F (1, 60) = 2.3248$, $p = 0.0303$). Random effect: mouse ID.

g. Fig.3C

Spatially distinct areas of non-injured (n = 20) and injured tubule segments (n = 38) from n = 5 male and 5 female partial IRI mice
Statistical test: linear mixed-effect model, response: PDGFRβ-cell area (% of total area), fixed effect: day ($F (9, 305) =17.55$, $p < 0.001$), group ($F (1, 305) = 0.095$, $p = 0.758$), day/group, ($F (9 , 305) =3.61$, $p < 0.001$). Random effect: mouse ID.

h. Fig. 3D

Binary regression analysis of tubule fate (0 = non-atrophy, 1 = atrophy) and the respective maximum tubule enclosure by PDGFRβ-cells before (grey curve) or after (red curve) the first detection of tubule injury (n = 512 tubules from 5 male and 5 female mice).
Equation: -3.632 + 0.074x. p (slope) < 0.001. R^2: 0.83. Odds Ratio: 1.086.

i. Fig. 3E

Binary regression analysis of tubule fate (0 = non-atrophy, 1 = atrophy) and the respective maximum tubule enclosure by PDGFRβ-cells before (grey curve) or after (red curve) the first detection of tubule injury (n = 512 tubules from 5 male and 5 female mice).
Equation: -0.1784 + 0.00785x. p (slope) = 0.54. R^2: 0.21. Odds Ratio: 1.00079.

j. Fig.3F

Binary regression analysis of tubule fate (0 = non-injury, 1 = injury) and the respective maximum tubule enclosure by PDGFRβ-cells before the first detection of tubule injury, if detectable (n = 470 tubules from 5 male and 5 female mice).
Equation: 0.530 - 0.033x. p (slope) < 0.001. R^2: 0.14. Odds Ratio: 0.96.

k. Fig.4B

Tubule NADH intensity (ex vivo D4). Mean ± 95% CI with scatterplot n = 209 tubule segments form 3 female mice.
Statistical test: linear mixed-effect model, response NADH intensity fixed effect: day, ($F (1, 207) = 227.09$, $p < 0.001$). Random effect: mouse ID.

l. Fig.4C
Tubule enclosure by PDGFRβ-cells (ex vivo D4). Mean ± 95% CI with scatterplot 209 tubule segments from 3 female mice.

Statistical test: linear mixed-effect model, response PDGFRβ-cell encrouchment, fixed effect: day, ($F (1, 207) = 228.4$, $p < 0.001$). Random effect: mouse ID.

m. Fig. 4E
Tubule NADH intensity (in vivo D4). Mean ± 95% CI with scatterplot n = 338 tubule segments form 3 male and 4 female mice.
Statistical test: linear mixed-effect model, response NADH intensity fixed effect: group, ($F (1, 336) = 5.6807$, $p < 0.0177$). Random effect: mouse ID.

n. Fig. 4F
Tubule enclosure by PDGFRβ-cells (in vivo D4). Mean ± 95% CI with scatterplot n = tubule segments form 3 male and 4 female mice.
Statistical test: linear mixed-effect model, response response PDGFRβ-cell encroachment, fixed effect: group, ($F (1, 319) = 6.9399$, $p < 0.008$). Random effect: mouse ID.

o. Fig. 4G

Logistic regression of injured tubule segments with or without dilated morphology. Outcome: tubule atrophy as binary classifier. Predictor: tubule morphology of injured tubule with categorical classification of non-dilated or dilated (n = 512 tubule segments from 5 male and 5 female mice).
Non-dilated: OR = 0.54, P = 0.29
Dilated: OR = 3.9, P < 0.001

p. Fig. 5C

Estimated time to 50% reduction in P-creatinine (days) vs. Vcam1 in biopsy. Multivariable linear regression of log values. Outcome: 50% reduction in P-creatinine (days). Predictor VCAM1 as % in biopsies (n = 21 males and 5 females). Covariants: sex and age.
$p = 0.001$. $F (3, 22) = 8.12$. adjusted $r2 = 0.46$. adjusted $r = 0.68$. $r2 = 0.53$
$tCr50 = 4.6 + 0.61 \times (Vcam1) - 0.0035 \times (Age) - 1.88 \times (Sex)$

q. Fig. 5D
eGFR 12 month vs. Vcam1 in biopsy.

Multivariable linear regression of log values. Outcome: eGFR 12 month (ml/min/1.73m2). Predictor VCAM1 as % in biopsies (n = 21 males and 5 females). Covariants: sex and age.
$p = 0.009$. $F (3, 22) = 3.43$. adjusted $r2 = 0.226$. adjusted $r = 0.475$. $r2 = 0.32$
GFR=68.48−8.21×(Vcam1)−0.104×(Age)-3.89×(Sex)

r. Fig. 5F

Estimated time to 50% reduction in P-creatinine (days) vs. KIM1 in biopsy.
Multivariable linear regression of log values. Outcome: 50% reduction in P-creatinine (days). Predictor KIM1 as % in biopsies (n = 18 males and 6 females). Covariants: sex and age.
$p = 0.535$. $F (3, 20) = 1.59$. adjusted $r2 = 0.07$. adjusted $r = 0.26$. $r2 = 0.19$
tCr50 =6.5 + 0.018×(KIM1)−0.031×(Age)-1.18×(Sex)

s. Fig. 5G

eGFR 12 month vs. Kim1 in biopsy.
Multivariable linear regression of log values. Outcome: eGFR 12 month (ml/min/1.73m2). Predictor KIM1 as % in biopsies (n = 18 males and 6 females). Covariants: sex and age.
$p = 0.366$. $F (3, 20) = 1.29$. adjusted $r2 = 0.036$. adjusted $r = 0.25$. $r2 = 0.16$
GFR=12.13−0.42×(VIM1)+0.74×(Age)-6.89×(Sex)

t. Fig. 5H

Estimated time to 50% reduction in P-creatinine (days) vs. Vcam1 in biopsy.
Multivariable linear regression of log values. Outcome: 50% reduction in P-creatinine (days). Predictor VCAM1 as % in biopsies (n = 30 males and 15 females). Covariants: sex and age.
$p = 0.002$. $F (3, 41) = 4.99$. adjusted $r2 = 0.21$. adjusted $r = 0.458$. $r2 = 0,27$
tCr50 =2.28 + 0.00009×(Vcam1)+0.011×(Age)-3.85×(Sex)

u. Fig. 5I

eGFR 12 month vs. day 5 plasma Vcam1.
Multivariable linear regression of log values. Outcome: eGFR 12 month (ml/min/1.73m2). Predictor day 5 plasma VCAM1 (pg/L). (n = 29 males and 15 females). Covariants: sex and age.
$p = 0.111$. $F (3, 40) = 2.31$. adjusted $r2 = 0.084$. adjusted $r = 0.289$. $r2 = 0.15$
GFR=37.88−0.0006×(Vcam1)−0.48×(Age)-12.14×(Sex)

Supplementary data

v. Fig. S1A

Partial IRI mice demonstrated albuminuria on day 1 after reperfusion, which thereafter reversed to control levels.

Statistical test: repeated measurement 2-way ANOVA, factors: treatment and days from treatment, post-hoc analysis: multiple comparisons, Bonferroni correction (n = 2 male and 1 female mice sham and 5 male and 4 female partial IRI mice).

Main fixed effect: Time x Treatment, p =0.03, F (7, 60) = 2,418
Multiple comparisons unique significant difference: day 1, t = 4.69, p <0,0001

w. Fig. S1B

No changes in GFR were detectable over time.
Statistical test: repeated measurement 2-way ANOVA, factors: treatment and days from treatment, post-hoc analysis: multiple comparisons, Bonferroni correction (n = 3 male and 2 female sham and 4 male and 4 female).
Main fixed effect: Time x Treatment, p =0.7096, F (7, 79) = 0,6544

x. Fig. S1C

Logistic regression of PI+ nuclei measured at day 0 after IRI (% of total nuclei) and probability of tubular atrophy (n = 512 tubules from 5 male and 5 female mice). r2 and odds ratio are reported as estimate of effect size. p-values from two-sided test. Predictor: PI+ nuclei %. Fit: log odds of predictor effect *x + log odds of intercept at predictor equal to zero.
Fit: 0.026x (p < .001, F (1,207) = 13.915) - 2.1603 (p < .001,F(1,207) = 36.005).
R^2: 0.066. Odds Ratio: 1.026 per unit of predictor.

y. Fig. S1D

No changes in PI were detected. Mean ± 95% CI with scatterplot. (n = 382 tubules) from 5 male and 5 female mice. Mean ± 95% CI with scatterplot.
Statistical test: linear mixed-effect model, response: GFP+ tubule cells (nuclei/mm3), fixed effect: group, (F (1, 379) = 2.65, p < 0.104). Random effect: mouse ID.

z. Fig. S1G

Albumin uptake in PT-S1 tubules.
Mean ± 95% CI with scatterplot. Sham (n = 97), undamaged (n = 41), recovering (n = 86) and atrophic S1 proximal tubules (n = 32) from 2 male and 1 female mice sham and 5 male and 5 female partial IRI mice.

Statistical test: linear mixed-effect model, response alexa594-albumin ratio (tubule/plasma), fixed effect: day (F (6, 1266) = 2.699, p = .013), group (F (3, 1266) = 9.611, p < .001), day/group (F (18, 1266) = 11.191, p < .001). Random effect: mouse ID.

aa. Fig. S2C

Mean ± 95% CI with scatterplot. n = 8 fields of view from 2 male and 1 female sham mice and n = 31 FOVs from 5 male and 5 female partial IRI mice.
Statistical test: linear mixed-effect model, response: PDGFRβ-cell area (% of total area), fixed effect: fixed effect: day (F (8, 214) = 0.686, p = .703), group (F (1, 214) = 0.462, p = .497), day/group (F (8, 214) = 4.6783, p < .001). Random effect: mouse ID.

bb. Fig. S3B

More proliferation is observed 3 days after IRI in the tubule.
Mean ± 95% CI with scatterplot. Control (n = 10 FOVs from 1 male and 6 female mice) and at days 3 (n = 13 FOVs from 1 male and 3 female mice) and 14 (n = 15 FOVs from 1 male and 3 female mice) post partial IRI
Statistical test: linear mixed-effect model, response: GFP+ tubule cells (nuclei/mm3), fixed effect: group, (F (2, 35) = 10.468, p < 0.001). Random effect: mouse ID.

cc. Fig. S3C

More proliferation is observed 3 days after IRI in PDGFRB cells.
Mean ± 95% CI with scatterplot. Control (n = 10 FOVs from 1 male and 6 female mice) and at days 3 (n = 13 FOVs from 1 male and 3 female mice) and 14 (n = 15 FOVs from 4 female mice) post partial IRI
Statistical test: linear mixed-effect model, response: GFP+ PDGFRβ+ cells (nuclei/mm3), fixed effect: group, (F (2, 35) = 22.187, p < 0.001). Random effect: mouse ID.

dd. Fig. 4A

There are more PDGFRB cells around damaged tubules.
Mean ± 95% CI with scatterplot. n = 229 tubule segments (no damage). n = 388 tubule segments (damage) from 5 male and 5 female partial IRI mice.
Statistical test: linear mixed-effect model, response: max tubular enclosure by PDGFRβ-cell area (%), fixed effect: group (F (1, 610) = 132.27, p < .001). Random effect: mouse ID.

ee. Fig. S5A, B

Mean ± 95% CI with scatterplot. n = 250 tubule segments from 2 male and 1 female mice mice), and partial IRI mice (n = 5 male and 5 female mice, categorized

based on outcomes: no damage (n = 225), recovery (n = 233), and atrophy (n = 142)
Statistical test: linear mixed-effect model, response: tubular enclosure by PDGFRβ-cells (%), fixed effect: day (F (9, 4083) =123.13, p < .001), group (F (2, 4083) = 108.59, p < .001), day/group (F (18, 4083) = 37,562, p < .001). Random effect: mouse ID.

ff. Fig. S6C

Quantification of overlap between αSMA and PDGFRβ signal from 3*3 tile scan areas from non-IRI areas (n = 6 from 3 male and 3 female mice), day 4 (n = 7 from 2 male and 2 female mice), day 7 (n = 6 from 1 male and 2 female mice) and day 21 from (n = 6 from 2 male and 1 female mice)
Statistical test: linear mixed-effect model, response: Overlap (% of total area), fixed effect: group (F (3, 20) = 27,6, p < 0.001). Random effect: mouse ID.

gg. Fig. S6D

Quantification of Sirius Red signal from from non-IRI areas (n = 27 from 4 male and 4 female mice), day 4 (n = 16 from 2 male and 2 female mice), day 7 (n = 15 from 1 male and 2 female mice) and day 21 from (n = 15 from 2 male and 1 female mice)
Statistical test: linear mixed-effect model, response: Sirius Red (% of total area), fixed effect: group (F (3, 69) = 76.065, p < 0.001). Random effect: mouse ID.

hh. Fig. S6G

Quantification of segmented SHG signal from sham (n = 6, 2 male and 1 female mice), day 7 remodeling (n = 14 from 2 male and 2 female mice), and day 20 recovered (n = 9) and atrophic areas (n = 7) after partial IRI (n = 1 male and 2 female mice).
Statistical test: linear mixed-effect model, response: SHG area (% of total area), fixed effect: group (F (3, 32) = 6,92, p = 0.001). Random effect: mouse ID.

ii. Fig. S6H

Quantification of segmented PDGFRβ signal from sham areas (n = 6, from 2 male and 1 female mice), day 7 remodeling (n = 14 from 2 male and 2 female mice), and day 20 recovered (n = 9) and atrophic areas (n = 7) after partial IRI (n = 1 male and 2 female mice).
Statistical test: linear mixed-effect model, response: PDGFRβ-cell area (% of total area), fixed effect: group (F (3, 31) = 28.495, p < .001). Random effect: mouse ID.

jj. Fig. S9B

Mean ± 95% CI with scatterplot. n = 4 field of views from 4 partial IRI from 1 male and 3 female mice.
Statistical test: linear mixed-effect model, response: wound size area (um2), fixed effect: day, ($F_{(8, 18)} = 4.3218$, $p = 0.0047$). Random effect: mouse ID.

kk. Fig. S10A, B

Spatially distinct areas of non-injured (n = 20) and injured tubule segments (n = 38) from 5 male and 5 female partial IRI mice
Statistical test: linear mixed-effect model, response: PDGFRβ-cell area (% of total area), fixed effect: day ($F_{(9, 305)} = 17.55$, $p < 0.001$), group ($F_{(1, 305)} = 0.095$, $p = 0.758$), day/group, ($F_{(9, 305)} = 3.61$, $p < 0.001$). Random effect: mouse ID.

ll. Fig.11C

Linear regression analysis of day 4 tubular NADH autofluorescence measure in vivo versus secondary AB intensity after ex vivo VCAM1 immunostaining (n = 209 tubule segments form 3 mice).

Statistical test: simple linear regression. $R^2 = 0.225$, $P < 0.001$, $r = 0.473$. Equation: Y = -0,6740*X + 1411

mm.Fig. S12A

Estimated time to 50% reduction in P-creatinine (days) vs. 5-day urine Vcam1.
Multivariable linear regression of log values. Outcome: 50% reduction in P-creatinine (days). Predictor VCAM1 as % in biopsies (n = 32 males/16 females).
Covariants: sex and age.
$p = 0.314$. $F_{(3, 44)} = 1.24$. adjusted $r^2 = 0.015$. adjusted $r = 0.122$
$tCr50 = 2.45 + 0.242\times(Vcam1)+0.014\times(Age)-0.61\times(Sex)$

nn. Fig. S12B

eGFR 12 month vs. 5-day urine Vcam1.
Multivariable linear regression of log values. Outcome: eGFR 12 month (ml/min/1.73m2). Predictor day 5 urine Urine Vcam1 (pg/L). (n = 31 males/16 females). Covariants: sex and age.
$p = 0.446$. $F_{(3, 43)} = 1.26$. adjusted $r^2 = 0.017$. adjsuted $r = 0.13$. $r^2 =$
$GFR=7.07 + 2.73\times(Vcam1)+0.397\times(Age)-7.796\times(Sex)$

oo. Fig. S13A

Boxplot of Vcam1 in day 6 graft biopsies transplanted patients (n= 21 males and 5 females) that were subjected to remote ischemic conditioning (n = 12) or no remote ischemic conditioning (n = 14). Two-sample t-test
$P = 0.056$

pp. Fig. S13B

Boxplot of Vcam1 in day 5 plasma of transplanted patients (n= 30 males and 15 females) that were subjected to remote ischemic conditioning (n = 23 or no remote ischemic conditioning (n = 22). Two-sample t-test
Day 5 plasma VCAM1 P = 0.51

qq. Fig. S13B

Boxplot of Vcam1 in day 5 urine of transplanted patients (n= 32 males and 16 females) that were subjected to remote ischemic conditioning (n = 24) or no remote ischemic conditioning (n = 24). Two-sample t-test
Day 5 plasma VCAM1 P = 0.445
